# Supplementary figures and images for: Five year mortality in an RCT of a lung cancer biomarker to select people for low dose CT screening
Source: PLoS One. 2025 Jan 8;20(1):e0306163. doi: 10.1371/journal.pone.0306163 (PMC11709295; doi:10.1371/journal.pone.0306163)

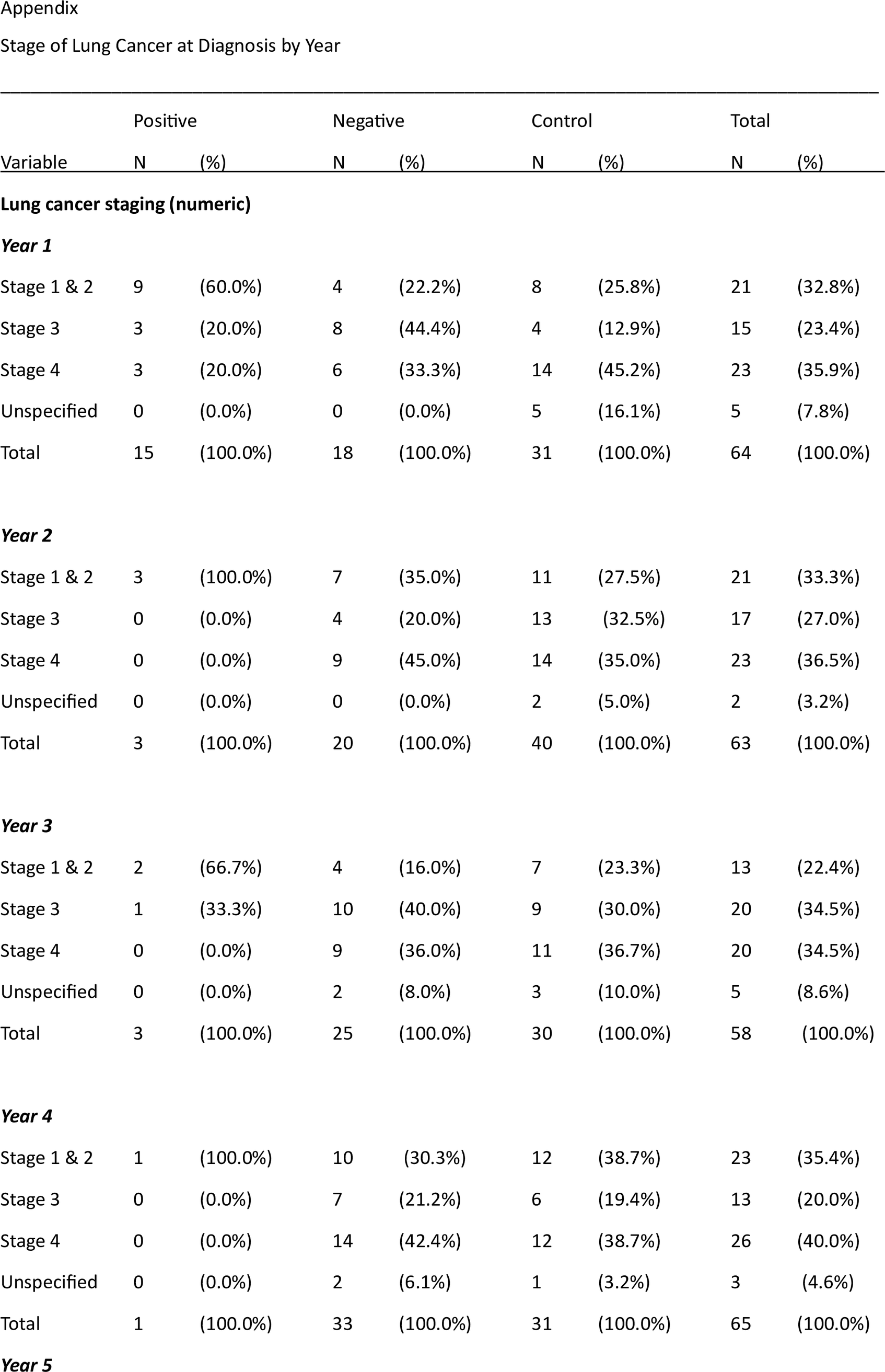

Supplement: S1 Appendix — (TIF) [file pone.0306163.s001.tif]
