# Supplementary material for: Five year mortality in an RCT of a lung cancer biomarker to select people for low dose CT screening
Source: PLoS One. 2025 Jan 8;20(1):e0306163. doi: 10.1371/journal.pone.0306163 (PMC11709295; doi:10.1371/journal.pone.0306163)
Supplement: S1 File — (PDF) [file pone.0306163.s002.pdf]

ECLS Study Protocol
Version 1.2 1st May 2013
Detection in blood of autoantibodies to tumour antigens as a case-finding method in lung cancer using the EarlyCDT-Lung test

**Study Acronym**	**ECLS**
**Sponsor**	University of Dundee - NHS Tayside
**Sponsor R&D Number**	2013ON07
**Coordinating Trial Centre**	Tayside CTU
**Funder**	Chief Scientist Office, Scottish Government<br>Oncimmune Ltd
**Chief Investigator**	Professor Frank Sullivan
**REC Number**	***13/ES/0024***
**ISRCTN Number**	
**Version Numbers and Dates**	Version 1.2, 1st May 2013

CONTACT NAMES AND ADDRESSES

Prof F M Sullivan (Chief Investigator, University of Dundee)
Division of Population Health Sciences, Kirsty Semple Way, Dundee, DD2 4BF
f.m.sullivan@dundee.ac.uk 01382 383738

Dr R Littleford (Senior Trial Manager, Tayside Clinical Trials Unit)
Tayside Clinical Trials Unit, Ninewells Hospital and Medical School, Dundee DD1 9SY
r.littleford@dundee.ac.uk 01382 383242

Cheryl Hume (Trial Administrator, Tayside Clinical Trials Unit)
Tayside Clinical Trials Unit, Ninewells Hospital and Medical School, Dundee DD1 9SY
c.l.hume@dundee.ac.uk 01382 383898

Primary and secondary objectives and outcomes: summary

	**Objectives**	**Outcomes**
**Primary**	to assess the effectiveness of EarlyCDT-Lung test in reducing the incidence of patients with late-stage lung cancer at diagnosis, compared with standard clinical practice;	difference at 24 months after randomisation, between the number of patients with stage 3, 4 or unclassified lung cancer at diagnosis in the intervention arm, and those in the control arm;
**Secondary 1**	to assess the effectiveness of EarlyCDT-Lung test in improving the diagnosis of early-stage lung cancers;	numbers, at 24months after randomisation, in the different stages at diagnosis (3/ 4/ U/ other) in the intervention arm and the control arm;
**2**	to undertake a cost-effectiveness analysis of EarlyCDT-Lung test as a primary screening method in comparison to standard clinical practice;	difference, after 2 years, in the costs and outcomes between the intervention arm and the control arm; cost-effectiveness of the intervention compared to standard clinical practice
**3a**	to compare lung-cancer mortality, all-cause mortality and cancer-specific mortality in high-risk groups provided with EarlyCDT-Lung test, compared with standard practice;	estimates, after 2 years, of lung cancer mortality, all-cause mortality and cancer-specific mortality in the intervention arm and in the control arm; assessment of significance of differences;
**3b**	to compare long-term future mortality in high-risk groups provided with EarlyCDT-Lung test, compared with standard practice;	estimates, after 5 and 10 years of long-term future mortality in the intervention arm and in the control arm; assessment of significance of differences;
**4**	to obtain refined estimates of the sensitivity, specificity, positive predictive value and negative predictive value of EarlyCDT-Lung test;	estimates, after 2 years of **(i)** the number of patients with stage 3, 4 or unclassified lung cancer at diagnosis in the EarlyCDT-Lung test-positive group and those in the EarlyCDT-Lung test-negative group and **(ii)** stage at diagnosis in the EarlyCDT-Lung test-positive and EarlyCDT-Lung test-negative group;
**5**	to assess behavioural outcomes including smoking, psychological outcomes including cancer worry, anxiety, depression, distress specific to clinical investigations;	scores at baseline, and follow-up on EQ5D, Hospital Anxiety and Depression Scale (HADS), Positive and Negative Affect Schedule (PANAS), Revised Illness Perception Questionnaire – Lung Cancer (IPQ-LC), Lung cancer risk perception, Health anxiety subscale of Health Orientation Scale (HOS) and the Lung Cancer Worry Scale (LCWS), Medication, smoking behaviour, demographic details. Follow-up questionnaires include same items, plus Impact of Events Scale (intervention group only), healthcare utilisation and dates and results of follow-up investigations for lung cancer (test positive group only). The HADS is not included in follow- up questionnaires. Follow-up questionnaires are administered between 1 and 24 months to subsets of the control arm and intervention arm;

		(all participants in the EarlyCDT-positive group will be approached with the recruitment aim of 300 from this group collected at 1,3,6,12,18 and 24 months. The EarlyCDT-negative and control groups will be recruited at the same rate as the EarlyCDT-positive group with the recruitment aim of 300 from each group collected at 1,3,6 and 12 months).
**6**	to assess the effectiveness of EarlyCDT-Lung test on other clinical outcomes such as CVD, COPD, other cancers, hospital stays and outcomes identified though SMR linkage, etc.;	Incidence of other clinical outcomes such as CVD, COPD, other cancers, hospital stays, identified through SMR linkage, measured at 24 months, 5 and 10 years in the intervention arm and in the control arm; assessment of significance of differences;
**7**	to assess uptake of subsequent investigations such as CXR, CT, bronchoscopy, etc.	numbers in all groups (EarlyCDT-Lung test-positive, EarlyCDT-Lung test-negative, control) undertaking subsequent investigations such as CXR, CT, bronchoscopy, etc.

CONTENTS

CONTENTS 4

PROTOCOL APPROVAL 6

LIST OF ABBREVIATIONS 9

SUMMARY 10

1 INTRODUCTION 17
1.1 BACKGROUND to the research 17
1.2 Research questions 19
1.3 RATIONALE FOR STUDY 20
1.3.1 Recruitment Strategy 21
1.3.2 Recruitment Materials 21
1.4 OBJECTIVEs 22
1.4.1 Primary Objective 22
1.4.2 Secondary Objectives 22
1.5 OUTCOMES 23
1.5.1 Primary Outcomes 23
1.5.2 Secondary Outcomes 23
2 STUDY DESIGN 25
2.1 STUDY DESCRIPTION 25
2.1.1 Setting 25
2.1.2 Participants 25
2.2 STUDY FLOWCHART 26
2.3 STUDY MATRIX 27
2.4 STUDY Assessment AND NOTIFICATIONS 28
3 STUDY POPULATION 29
3.1 NUMBER OF PARTICIPANTS 29
3.2 INCLUSION CRITERIA 29
3.3 EXCLUSION CRITERIA 30
4 PARTICIPANT SELECTION AND ENROLMENT 31
4.1 IDENTIFYING PARTICIPANTS 31
4.2 CONSENTING PARTICIPANTS 34
4.3 SCREENING FOR ELIGIBILITY 35
4.4 INELIGIBLE AND NON-RECRUITED PARTICIPANTS 35
4.5 RANDOMISATION 35
4.6 Administration of the test 36
4.7 Management of the visits 36
4.8 Withdrawal and study termination procedures 37
5 STUDY AND SAFETY ASSESSMENTS STUDY ASSESSMENTS 37
5.1 SAFETY ASSESSMENTS 38
6 DATA COLLECTION & MANAGEMENT 38
6.1 Data Collection 38
6.2 Data Management System 40
7 STATISTICS AND DATA ANALYSIS 41
7.1 SAMPLE SIZE CALCULATION 41
7.2 PROPOSED ANALYSES 43
7.2.1 Cost effective analysis 43
7.3 Missing data 44
7.4 TRANSFER OF DATA 44
7.5 PREGNANCY 44

8 TRIAL MANAGEMENT AND OVERSIGHT ARRANGEMENTS ... 44
8.1 TRIAL MANAGEMENT GROUP ... 44
8.2 TRIAL MANAGEMENT ... 45
8.3 TRIAL STEERING COMMITTEE ... 45
8.4 DATA MONITORING COMMITTEE ... 45
8.5 INSPECTION OF RECORDS ... 46
8.6 RISK ASSESSMENT ... 46
8.7 STUDY MONITORING ... 46
8.7.1 Potential Risks ... 46
8.7.2 Blood sampling ... 46
8.7.3 Test results ... 47
8.7.4 Radiography ... 47
8.7.5 Minimising Risk ... 48
9 GOOD CLINICAL PRACTICE ... 48
9.1 ETHICAL CONDUCT OF THE STUDY ... 48
9.1.1 Confidentiality ... 48
9.1.2 Data Protection ... 48
9.1.3 Insurance and Indemnity ... 49
10 STUDY CONDUCT RESPONSIBILITIES ... 50
10.1 PROTOCOL AMENDMENTS, DEVIATIONS AND BREACHES ... 50
10.2 STUDY RECORD RETENTION ... 50
10.3 END OF STUDY ... 51
10.4 CONTINUATION OF TREATMENT FOLLOWING THE END OF STUDY ... 51
11 REPORTING, PUBLICATIONS AND NOTIFICATION OF RESULTS ... 51
11.1 AUTHORSHIP POLICY ... 51
11.2 PUBLICATION ... 52
11.3 PEER REVIEW ... 52
12 REFERENCES/BIBILOGRAPHY ... 53
APPENDIX 1: PREPARATORY FOCUS GROUP WORK ... 56

1.1 Protocol ... 56

1.2 APPROVAL ... 59

1.3 FINAL REPORT ... 60

APPENDIX 2. Trial Steering Committee ... 60

APPENDIX 3. Data Monitoring Committee ... 64

PROTOCOL APPROVAL

Title: Detection in blood of autoantibodies to tumour antigens as a case-finding method in lung cancer using the EarlyCDT-Lung test

ISRCTN Reference

Signatures

By signing this document I am confirming that I have read, understood and approve the protocol for the above study.

**Chief Investigator**	**Signature**	**Date**
Prof F M Sullivan<br>University of Dundee		
**Co-Investigators**	**Signature**	**Date**
Prof John Robertson<br>University of Nottingham		
Prof Kavita Vedhara<br>University of Nottingham		
Prof Denise Kendrick<br>University of Nottingham		
Prof Herb Sewell<br>University of Nottingham		
Dr Alistair Dorward<br>NHS Greater Glasgow & Clyde		
Prof Shaun Treweek<br>University of Aberdeen		
Dr Colin McCowan<br>University of Glasgow		
Prof Chris Robertson<br>University of Strathclyde		
Dr Mike Sproule<br>NHS Greater Glasgow & Clyde		
Dr Elisabeth Fenwick<br>University of Glasgow		
Prof Frances Mair<br>University of Glasgow		
Prof Lewis Ritchie<br>University of Aberdeen		
Dr Stuart Schembri<br>University of Dundee		
Philippe Autier<br>International Prevention<br>Research Institute		

Dr Robert Milroy<br><br>NHS Greater Glasgow & Clyde		
Dr Tom Taylor<br><br>NHS Tayside		
Dr Roberta Littleford<br><br>TCTU, University of Dundee		

**Local Investigators**	**Signature**	**Date**
Dr William Anderson<br>NHS Tayside		
Dr Peter Brown<br>NHS Tayside		
Dr Roddie Cameron<br>NHS Tayside		
Dr Alan Cook<br>NHS Tayside		
Dr Scott Davidson<br>NHS Greater Glasgow & Clyde		
Dr Tom Fardon<br>NHS Tayside		
Dr Peter Garmany<br>NHS Greater Glasgow & Clyde		
DrYoris van der Horst<br>NHS Greater Glasgow & Clyde		
Dr Ewan Ross<br>NHS Greater Glasgow & Clyde		
Dr Joseph Sarvesvaran<br>NHS Greater Glasgow & Clyde		
Dr Simon Sheridan<br>NHS Greater Glasgow & Clyde		
Dr Robin Smith<br>NHS Tayside		
Dr Tom Taylor<br>NHS Tayside		

**Collaborators**	**Signature**	**Date**
Dr David Brewster<br>Scottish Cancer Registry		
Dr Paul Sergeant<br>Tay Dynamic Ltd		

**Statistical Review**	**Signature**	**Date**
Dr Petra Rauchhaus<br>Tayside CTU		

LIST OF ABBREVIATIONS

AAB	Autoantibody
AE	Adverse event
CRF	Care Report Form
CI	Chief Investigator
CNORIS	clinical negligence and other risks scheme
CT scan	Computerised Tomography Scan
CXR	Chest X-Ray
eCRF	Electronic case report form
EarlyCDT – Lung Test	Early Cancer Detection Test- Lung Test
ECLS Study	Early Cancer Detection Test – Lung Cancer Scotland Study
GG&C	Greater Glasgow & Clyde
GCP	Good Clinical Practice
HIC	Health Informatics Centre
ICF	Informed Consent Form
ISF	Investigator Site File
SAE	Serious adverse event
SCR	Scottish Cancer Register
SMR	Scottish Morbidity Record
SOP	Standard Operating Procedure
TASC	Tayside Medical Science Centre
TCTU	Tayside Clinical Trials Unit
TAA	Tumour Derived/Associated Antigens
TMF	Trial Master File

SUMMARY

QUESTION / RATIONALE

Lung cancer is the most common cause of cancer-related death worldwide. The majority of cases are detected at a late stage when prognosis is poor. Lung cancer remains the fourth least likely cancer to be picked up early by GPs. Low dose computed tomography (CT) scanning of high risk individuals can reduce lung cancer mortality by 20% but it is expensive and, despite scanning, late stage diagnosis results in substantial morbidity.

The EarlyCDT-Lung Test is an early detection test designed to assist in lung cancer risk assessment and detection in the earliest stages of the disease. Survival rates are much higher when cancer is diagnosed early but because lung cancer is often diagnosed symptomatically, most cases are discovered after the disease has spread. In these cases, the 5-year survival rate is less than 10%. By testing patients who are at a high risk for developing lung cancer before symptoms appear, the EarlyCDT-Lung test could help diagnose lung cancer sooner, when treatment options are more likely to be successful. The EarlyCDT-Lung test detects autoantibodies, which are a patient’s immune response to antigens produced by solid-tumor cells. Because these autoantibodies are produced by healthy individuals at lower levels, the EarlyCDT-Lung test enables physicians to identify those patients producing autoantibodies at higher levels and who are at an increased lung cancer risk or who are already in the early stages of lung cancer.

The EarlyCDT-Lung test can potentially identify those at high risk of lung cancer in whom the benefit/risk ratio for CT scanning is likely to be more favourable. The primary research question is therefore:

Does using the EarlyCDT-Lung test to identify those at high risk of lung cancer and any subsequent CT scanning reduce the incidence of patients with late-stage lung cancer (3 & 4) or unclassified presentation (U) at diagnosis, compared with standard practice?

Secondary questions include, but are not limited to:

i) Is the use of the EarlyCDT-Lung test cost-effective compared to standard clinical practice?

ii) What is the short and long term emotional and behavioural impact of the EarlyCDT-lung test?

iii) Does the EarlyCDT-Lung test improve clinical outcomes including but not limited to cardiovascular disease (CVD), COPD, hospital stays and outcomes identified through SMR linkage?

HYPOTHESIS

In a high risk population the EarlyCDT-Lung test reduces the incidence of late stage tumours;3 / 4 / Unclassified (U) at diagnosis compared to normal clinical practice.

AIMS

To assess the effectiveness of EarlyCDT-Lung test in increasing early stage lung cancer detection, thereby reducing the rate of late stage (3 / 4 / U) presentation, compared to normal clinical practice;

to assess the cost-effectiveness of EarlyCDT-Lung test compared to normal clinical practice;

to assess the effectiveness of EarlyCDT-Lung test in reducing adverse outcomes including potential psychological and behavioural consequences.

DESIGN

We propose a randomised controlled trial of 10,000 participants. Cancer screening

programmes should be based on the high quality evidence which trials provide that they reduce cancer specific mortality. People should be invited to participate in population screening programmes on the basis of firm evidence that the overall balance between potential benefits and harms is favourable. Where screening programmes have relied upon observational data, for example in breast and prostate cancer screening programmes have remained controversial for many years. Eventually in the case of breast cancer large trials have been undertaken to determine the place of the screening method in national programmes. In contrast where large trials have preceded regional and national roll-out of cancer screening programmes e.g. in bowel cancer, the programmes have been more evidence based (for example, population based trials of faecal occult blood testing have consistently demonstrated significant reductions in colorectal cancer mortality and are summarised in a meta-analysis that indicates a reduction of 16% overall and 25% when adjusted for screening uptake). In the case of lung cancer we have observational data to suggest that the Early CDT-Lung test may be effective and it is now necessary to undertake a trial to determine whether this potential benefit outweighs potential harms and whether the test would be a cost effective use of NHS resources.

SETTING

Initially, we plan to recruit participants via 60-80 general practices, predominately within the lowest quintile of deprivation measured using the Scottish Index of Multiple Deprivation (from a total of 200 in that quintile) in NHS Tayside and NHS Greater Glasgow & Clyde (GG&C). However, it is anticipated that a number of potential participants will contact the study team in response to the initial media interest surrounding the launch of the study and via family and friends of randomised participants. All interested individuals out with the GP recruitment strategy will be assessed in relation to inclusion/exclusion criteria including residing within the

selected geographical post codes. These participants will be screened at either their participating GP practice or at the local Clinical Research Facility/Centre.

PARTICIPANTS

Adults aged 50 to 75 who are at risk of lung cancer will be eligible to participate. These are defined as those who are current or former cigarette smokers with at least 20 pack-years, or have a history of cigarette smoking less than 20 pack-years plus a family history (mother, father, brother, sister) of lung cancer which gives an individual a personal risk similar to a smoking history of 20 pack years. Participants should be healthy enough to undergo pulmonary resection or stereotactic radiotherapy.

INTERVENTION

EarlyCDT-Lung test followed by imaging studies in those with a positive result.

COMPARATOR

Standard practice of awaiting clinical presentation of symptoms suggestive of lung cancer then investigation by the standard NHS pathway involving chest X-ray, CT scan and bronchoscopy as clinically necessary.

OUTCOMES

Primary

The difference, at 24 months after randomisation, between the rates of patients with stage 3, 4 or unclassified lung cancer at diagnosis in the intervention arm, and those in the control arm;

Secondary

(1) numbers at 24 months after randomisation, in the different stages at diagnosis (3/ 4/ U/ other) in the intervention arm and the control arm;

(2) difference, after 2 years, between costs and outcomes in the intervention arm and in the control arm, cost-effectiveness of EarlyCDT-Lung test compared to normal clinical practice;

(3a) estimates, after 2 years, of lung cancer mortality, all-cause mortality and cancer-specific mortality rates in the intervention arm and in the control arm ; assessment of significance of differences;

(3b) estimates, after 5 years and 10 years, of long-term future mortality rates in the intervention arm and in the control arm; assessment of significance of differences;

(4) estimates, after 2 years of, provided by (i) the number of patients with stage 3, 4 or unclassified lung cancer at diagnosis in the EarlyCDT-Lung test-positive group and those in the EarlyCDT-Lung test-negative group and (ii) stage at diagnosis in the EarlyCDT-Lung test-positive and EarlyCDT-Lung test-negative group;

(5) scores at baseline, and follow-up on in a survey administered prior to treatment allocation, including EQ5D, Hospital Anxiety and Depression Scale (HADS), Positive and Negative Affect Schedule (PANAS), Revised Illness Perception Questionnaire – Lung Cancer (IPQ-LC), Lung cancer risk perception, Health anxiety subscale of Health Orientation Scale (HOS) and the Lung Cancer Worry Scale (LCWS), Medication, smoking behaviour, demographic details. Follow-up questionnaires include same items, plus Impact of Events Scale (intervention group only) and healthcare utilisation. The HADS is not included in follow- up questionnaires. Follow-up questionnaires are EQ-5D, cancer worry, positive and negative mood, smoking behaviour including cessation intentions and attempts; scores in additional questionnaires administered at between 1 and 24 months to subsets of the control arm and intervention arm; (all participants in the EarlyCDT-positive group will be approached with the recruitment aim of 300 from this group collected at 1,3,6,12,18 and 24 months. The EarlyCDT-negative and control groups will be recruited at the

same rate as the EarlyCDT-positive group with the recruitment aim of 300 from each group collected at 1,3,6 and 12 months)..

(6) incidence at 24 months, and after 5 years and 10 years, in other clinical measures such as CVD, COPD, hospital stays, and outcomes identified through SMR linkage, etc. in the intervention arm and in the control arm; assessment of significance of differences;

(7) numbers in all groups (EarlyCDT-Lung test-positive, EarlyCDT-Lung test-negative, control) undertaking subsequent investigations such as CXR, CT, bronchoscopy, etc.

METHODS

Based on the test's 93% specificity and 41% sensitivity we anticipate that 400-450 participants in the intervention arm will have a positive test result. These will be offered a chest X-ray. Those with a negative or indeterminate X-ray will be referred for a study CT scan. If the initial CT is negative then subsequent CTs will be offered 6 monthly for 24 months. Those Individuals with monitorable abnormalities as classified by the radiology/respiratory physician's study panel on baseline CT scan or subsequent CT will be followed up over the study period or referred for NHS clinical care as appropriate. All individuals entering the study will be flagged and followed up via the Scottish Cancer Registry. Participants who develop lung cancer will be individually followed-up via electronic record-linkage to assess both time to diagnosis and stage of disease at diagnosis. If no histological stage is available, stage will be assessed blind to allocation status from chest X-rays or CT, or, if no imaging is available a medical assessment of stage will be carried out.

HOW THE RESULTS OF THIS RESEARCH WILL BE USED

The study will assess the EarlyCDT-Lung test's clinical and cost effectiveness and suitability for a large-scale, accredited screening service for early lung cancer detection. It will also assess potential morbidity arising from the test and potential harms and benefits of a negative EarlyCDT-Lung test result.

DATES AND DURATION OF TRIAL

01/04/2013 – 30/09/16 (42 months)

1 INTRODUCTION

1.1 BACKGROUND TO THE RESEARCH

Lung cancer is the world's leading cause of cancer related mortality and a major source of morbidity. 85% of patients with lung cancer remain undiagnosed until the disease is symptomatic and has reached an advanced stage. Moreover, Scotland has had one of the highest rates of lung cancer in the world. Around 2,460 men and 2,340 women are diagnosed with lung cancer in Scotland every year, which is 16% of the total UK lung cancer cases, despite Scotland having 8% of the UK's population. Survival from lung cancer is poor with less than 9% of patients still alive at five years after diagnosis, due primarily to late stage of presentation.

Early detection and diagnosis of cancer improves prognosis - the current 5-year survival rate is approximately 60% for stage I lung cancer but is only 1% for those with stage IV disease. The potential of early detection of lung cancer to improve outcomes was highlighted by the National Cancer Institute (NCI) National Lung Screening Trial (NLST) which recently reported that CT screening reduced lung cancer mortality by 20%. However as a primary screening modality CT is expensive and leads to substantial morbidity in a significant percentage of individuals whose tests are false positives. The EarlyCDT-Lung test is an innovative diagnostic test for early detection of lung cancer. The test can stratify individuals by risk of developing future lung cancer; those with a positive test are invited for a chest X-ray then, if that is normal, a CT scan. This targeted approach to CT scanning for early lung cancer detection is likely to be a more cost-effective and potentially less harmful approach to population screening than a blanket CT-scanning program of all people considered at high risk of future lung cancer.

A substantial body of published research has documented autoantibody (AAB) responses against various tumour derived/associated antigens (TAA) in patients with

a wide range of solid tumours, including lung cancer. The serum proteome provides an attractive source of potential biomarkers and because serum collection is minimally invasive it can be repeatedly surveyed for cancer biomarkers. AABs have been detected months to years before clinical diagnosis of breast and lung cancers, supporting the hypothesis that AABs could be incorporated into an early detection assay. Subsequent research studies have confirmed AABs to TAAs in patients with early stage lung cancer. AABs have been reported in lung cancer subjects up to 5 years before clinical diagnosis even where annual screening spiral CTs were being performed.

A serum assay has been developed and validated called Early Cancer Detection Test-Lung (EarlyCDT-Lung) that can detect 40% of lung cancers with a specificity of 90% by measuring autoantibodies to a panel of cancer antigens (p53, NY-ESO-1, CAGE, GBU4-5, Annexin1, & SOX2). Further confirmation of this sensitivity and specificity of the test for lung cancer using four new, independent sample sets has recently been published. A study of patient demographics showed no difference in autoantibodies based on age, gender and ethnicity. This autoantibody technology is different from CT scanning which in a prevalence screening test has a sensitivity of 67% for lung cancers developing over the following 12 months but with a low specificity of only around 49%. Indeed a prevalence CT screen will detect approximately 36% of the lung cancers which will develop in the next three years. If the EarlyCDT-Lung test has a three year ‘look forward’, as clinical data suggests, then the test will detect 40% of lung cancers which develop over this three year time period but with seven times fewer false positives than CT scanning. Two new autoantibodies (AAbs) have recently been added to the panel (and one removed) and the test now measures seven; p53, NY-ESO-1, CAGE, GBU4-5, HuD, MAGE A4 & SOX2 and identifies 41% of lung cancers with an increased specificity of 93% (Chapman et al; 2012). The 7-AAbs panel will be utilised in this study and all

statistical calculations are based on the 41% sensitivity and 93% specificity of the Early CDT-Lung test.

EarlyCDT-Lung detects lung cancer at all stages – i.e. it detects early stage lung cancer as well as advanced disease - which means autoantibodies are present at all stages of disease. In a large group of patients with newly diagnosed lung cancers there was no difference in positivity rate for EarlyCDT-Lung in early or late stage disease lung cancers – whether this was looking at all lung cancers, only non small-cell (NSC) lung cancer, NSC lung cancer, or only small-cell lung cancer (SCLC). Thus, while autoantibodies are present in early stage they are not a biomarker of only early stage disease. An audit, (presented July 2011 at the International Association for The Study of Lung Cancer) of the first 1000 patients to take the EarlyCDT-Lung test commercially, further confirms that the test works in clinical practice. These data are promising but an insufficient basis for introducing a national lung cancer screening program in the UK.

1.2 RESEARCH QUESTIONS

The primary research question is:

Does using the EarlyCDT-Lung test to identify those at high risk of lung cancer and any subsequent CT scanning reduce the incidence of patients with late-stage lung cancer (3 & 4) or unclassified presentation (U) at diagnosis, compared with standard practice?

Secondary questions include, but are not limited to:

i) is the use of the EarlyCDT-Lung test cost-effective compared to standard clinical practice?

ii) what is the emotional and behavioural impact of the EarlyCDT-lung test?

iii) does the EarlyCDT-Lung test improve clinical outcomes including but not limited to CVD, COPD, other cancers, hospital stays, outcomes identified through SMR linkage, etc.?

1.3 RATIONALE FOR STUDY

Lung cancer is the most common cause of cancer related death worldwide. The majority of cases are detected at a late stage when prognosis is poor.

CT scanning can reduce lung cancer mortality by 20%, but there are too many false positives leading to a large number of individuals without cancer being exposed to repeated unnecessary radiation.

A disproportionate amount of patients are given cause for concern when between 35%-75% of patients screened by CT are treated as positive (with resultant increased anxiety) but only 2%-3% will have a true cancer.

Active interventions (e.g. trans-thoracic biopsy, surgical resection) as the result of positive CT scans give rise to significant side effects and complications in a percentage of individuals.

The cost of screening with CT is expensive and unlikely to meet the thresholds for cost-effectiveness (£20k - £30k/QALY) usually used within the UK by bodies such as NICE.

Background to the study: pre-trial qualitative work

Four focus group sessions (Ethical approval by I-WHO, University of Nottingham, Appendix 1) were held with smokers aged 50 and over living in some of Glasgow and Dundee’s most deprived areas in order to explore recruitment preferences and likely willingness to participate in the forthcoming EarlyCDT Lung Cancer Scotland (ECLS) Study.

The work was carried out throughout June and July 2012 in four areas of Scotland: Castlemilk, Darnley, Charleston and Douglas. A total of 32 people aged 50 – 75 took part in the work, including 14 men and 18 women. All but one were current smokers, and most had smoked for 40 years or more, smoking one pack or more per day.

The findings from the work enabled the formation of a number of recommendations for both the main trial recruitment strategy and materials, including:

1.3.1 Recruitment Strategy

- Adopting a personal approach to invitations, sent from GPs and followed up in writing;
- Setting deadlines for people to respond to invitations to maximise likely response rates, bolstered by local radio and newspaper coverage of the study;
- Providing early summary information which emphasises that the study is not focussed on trying to encourage people to stop smoking;
- Telling people which group they are in *after* taking their blood in order to minimise attrition during initial appointments but also emphasising the value of being in the 'non-test group' for the benefit of wider research and public health; and
- Offering flexible appointments that are close to people's homes.

1.3.2 Recruitment Materials

- Making sure that all documents explicitly say that the trial relates solely to lung cancer;
- Explaining the reasoning for the study design (including control and intervention groups) and setting out clearly what the inclusion/exclusion criteria are, and why these criteria apply;
- Explaining the purpose of randomisation, and ensuring early on that people know when they will be notified of which group they are in. This includes

offering assurances that random means random and that being placed in the test group is not an indicator of risk;

- Acknowledging that not only smokers can be affected by lung cancer; and
- Offering sufficient information on the issue of making blood available to other researchers, and what this might entail, to allow fully informed consent to be given.

Trial documents have been developed based on the learning to emerge from these groups which will hopefully maximise participation in the upcoming trial and forearm those involved in its delivery as to the potential barriers to participation that may exist among the target population.

1.4 OBJECTIVES

1.4.1 Primary Objective

To assess the effectiveness of EarlyCDT-Lung test in reducing the incidence of patients with late-stage lung cancer at diagnosis compared with standard practice.

1.4.2 Secondary Objectives

1) to assess the effectiveness of EarlyCDT-Lung test in improving the diagnosis of early-stage lung cancers;

2) to undertake a cost-effectiveness analysis of EarlyCDT-Lung test as a primary screening method compared to standard clinical practice;

3a) to compare lung-cancer mortality, all-cause mortality and cancer-specific mortality in high-risk groups provided with EarlyCDT-Lung test, compared with standard practice;

3b) to compare long-term future mortality in high-risk groups provided with EarlyCDT-Lung test, compared with standard practice;

4) to obtain refined estimates of the sensitivity, specificity, positive predictive value and negative predictive value of EarlyCDT-Lung test;

5) to assess behavioural outcomes including smoking, psychological outcomes including cancer worry, anxiety, depression, distress specific to clinical investigations;

6) to assess the effectiveness of EarlyCDT-Lung test on other clinical outcomes;

7) to assess uptake of subsequent investigations.

1.5 OUTCOMES

1.5.1 Primary Outcomes

The difference, at 24 months after randomisation, between the number of patients with stage 3, 4 or unclassified lung cancer at diagnosis in the intervention arm, and those in the control arm;

1.5.2 Secondary Outcomes

1) numbers, at 24 months after randomisation, in the different stages at diagnosis (3/ 4/ U/ other) in the intervention arm and the control arm;

2) difference, after 2 years, between costs and outcomes in the intervention arm and in the control arm, cost-effectiveness of EarlyCDT-Lung test compared to normal clinical practice;

3a) estimates, after 2 years, of lung cancer mortality, all-cause mortality and cancer-specific mortality rates in the intervention arm and in the control arm; assessment of significance of differences;

3b) estimates, after 5 years and 10 years of long-term future mortality rates in the intervention arm and in the control arm; assessment of significance of differences;

4) estimates, after 2 years of (i) the number of patients with stage 3, 4 or unclassified lung cancer at diagnosis in the EarlyCDT-Lung test-positive group and those in the

EarlyCDT-Lung test-negative group and (ii) stage at diagnosis in the EarlyCDT-Lung test-positive and EarlyCDT-Lung test-negative group;

5) scores at baseline, and follow-up on in a survey administered prior to treatment allocation, including EQ5D, , Positive and Negative Affect Schedule (PANAS), Revised Illness Perception Questionnaire – Lung Cancer (IPQ-LC), Lung cancer risk perception, Health anxiety subscale of Health Orientation Scale (HOS) and the Lung Cancer Worry Scale (LCWS), Medication, smoking behaviour, demographic details. Follow-up questionnaires include same items, plus Impact of Events Scale (intervention group only), healthcare utilisation and dates and results of follow-up investigations for lung cancer (test positive group only). The HADS is not included in follow- up questionnaires. Follow-up questionnaires are EQ-5D,cancer worry, positive and negative mood, smoking behaviour including cessation intentions and attempts; scores in additional questionnaires administered at between 1 and 24 months to subsets of the control arm and intervention arm; (all participants in the EarlyCDT-positive group will be approached with the recruitment aim of 300 from this group collected at 1,3,6,12,18 and 24 months. The EarlyCDT-negative and control groups will be recruited at the same rate as the EarlyCDT-positive group with the recruitment aim of 300 from each group collected at 1,3,6 and 12 months).

6) incidence at baseline, 24 months, and after 5 years and 10 years, in other clinical measures such as CVD, COPD, other cancers, hospital stays, and outcomes identified through SMR linkage, etc. in the intervention arm and in the control arm; assessment of significance of differences;

7) numbers in all groups (EarlyCDT-Lung test-positive, EarlyCDT-Lung test-negative, control) undertaking subsequent investigations such as CXR, CT, bronchoscopy, etc.

2 STUDY DESIGN

2.1 STUDY DESCRIPTION

A randomised controlled trial involving 10,000 participants recruited through primary care and community based recruitment strategies in Scotland.

2.1.1 Setting

Initially, 60-80 general practices in the lowest quintile of deprivation in Scotland (from a total of 200 in that quintile) as measured by the quintiles of the Scottish Index of Multiple Deprivation (SIMD 2012). Subsequent recruitment will be attained through adverts, posters, flyers and community based interactions. Potential participants can either be seen at their participating GP practice or at the local clinical research centre, or other appropriate clinical location.

2.1.2 Participants

Adults aged 50 to 75 who are at risk of lung cancer will be eligible to participate. These are defined as those who are, current or former cigarette smokers with at least 20 pack-years, or have a history of cigarette smoking less than 20 pack-years plus an immediate family history (mother, father, brother, sister, child) of lung cancer which gives an individual a personal risk similar to a smoking history of 20 pack years. Participants should be healthy enough to undergo pulmonary resection or stereotactic radiotherapy.

2.2 STUDY FLOWCHART

GP Invitation Letter/Study Introduction via alternative Recruitment Strategy

POSITIVE RESPONSE

- Send/email full PIS/PIB to non-GP recruitment strategy participants
- Make or receive call from participant
- Discuss study/check screening eligibility & arrange appointment
- Sent/email appointment letter

(all participants have opportunity to cancel appointment after reading full PIS)

VISIT 1 @ GP PRACTICE OR CLINICAL RESEARCH FACILITY (DUNDEE/GLASGOW) ~30-45MINS

- See Research Nurse (RN) and obtain Informed Consent
- Check inclusion/exclusion criteria
- Obtain 10ml blood sample (for EarlyCDT test (0.5mls)/cancer related medical research)
- invite to complete study questionnaire (with assistance, if required)
- Web based randomisation – group allocation
- Remind participants that they will receive results in 4 weeks (test group only)

POST BLOODS TO UNIVERSITY OF NOTTINGHAM (UON)

- UoN process 10ml sample and send 0.5ml of test group sample to Omcimmune, Kansas, USA and store test and non-test blood for future cancer related medical research (with consent)

EARLYCDT TEST RESULTS

RN obtains results via secure portal within 4 weeks of Visit 1

POSITIVE RESULT

Letter sent inviting participant to attend/call
Visit 2 @ GP/CRF (~30 -45 mins)

- CXR/Study CT arranged
- Summary information given/posted to Participant (PIS 2)
- Participation & Results Letter sent to GP

NEGATIVE RESULT

- Results letter sent /offer to discuss with RN

6 MONTHLY STUDY CT SCANS

- Participant given appointments to attend for Study CT scans every 6 months for 2 years. Research team call 2-4 days before CT appt.

CXR & STUDY CT SCAN @ LOCAL HOSPITAL

- Participant attends for CXR
- If CXR negative participant attends for CT scan
- If CXR positive or suspicious participant informed to attend for NHS CT scan and seen by PI (Respiratory Physician) if required

(if NHS pathway negative, participant remains in study pathway)

CLINICALLY SIGNIFICANT CXR/CT SCAN/ IMAGE INCIDENTAL FINDINGS

Results deemed clinically significant by review panel of radiologists/respiratory physicians or GPs will be followed up via NHS routine clinical care. ***Mortality/Morbidity/hospitalisations collected as outcome data for ALL PARTCIPANTS.***

2.3 STUDY MATRIX

Recruitment Via GP Practices

Identify 200 GP practices out of approx 1000 (i.e. Q1, bottom quintile)

Of these 150 are in Tayside / GCC

Of these approach 80 – 100

Of these recruit 60 – 80

At each practice:

Identify patients satisfying Inclusion/Exclusion criteria

Approach 200 – 250 eligible patients / practice

Recruit 130 – 170 patients / practice

(60 – 80) practices x (130 – 170) patients/ practice ≈ 10,000 patients

Recruits, visits, samples

10,000 (recruits, visits, samples)

in 10 months @ 4 days/ week @ 48 weeks/ year

= 10,000 (recruits, visits samples) in 160 days

≈ 63 (recruits, visits, samples) per day or 250 / week

2.4 STUDY ASSESSMENT AND NOTIFICATIONS

Table 1. Overview of Study Assessments/Notifications.

ASSESSMENT/PROCEDURES	TIMELINE* (± 2 weeks)			
	Visit 1 (~30-45mins)		Visit 2* (~30mns)	➢ **EarlyCDT Positive Test Participants may visit or call.* ➢ *EARLY CDT Negative Test Participants may attend for further information/advice only.*
Informed Consent	**X**			
Inclusion/Exclusion Criteria	**X**			
• *Review/Record only Relevant Medical History relating to IC/EC*	**X**			
• *Review/Record Relevant Medications* • *Relating to IC/EC*	**X**			
Blood Sample	**X**			
Baseline Questionnaire	**X**			
Thank you letter to Control Group		**X**		
EarlyCDT- Lung Test Result Letter		**X**		
GP Results Letter & ICF copy (negative)		**X**		
Result Discussion/ Imaging Schedule			**X**	
Provide PIS 2			**X**	
GP Result Letter & ICF copy (positive)			**X**	

**EARLYCDT – Lung Test Positive Result Participants – Imaging Schedule**					
	TIMELINE(± 4 weeks)				
	0	6 months	12 months	18 months	24 months
CXR	**X**				
*CT Scan**	**X**	**X**	**X**	**X**	**X**
****Scheduled every 6 months, if participant enters NHS clinical care pathway, subsequent study CTscans will be cancelled.***		***Research team member will call 2-4 days before each scheduled CT scan to check health status and attendance.***			

3 STUDY POPULATION

3.1 NUMBER OF PARTICIPANTS

Approximately 170 participants from each of 60-80 general practices in the most deprived quintile of the population Scotland (from a total of 200 in that quintile) as measured by the quintiles of the Scottish Index of Multiple Deprivation (SIMD) 2012 - version 2.

3.2 INCLUSION CRITERIA

1. Participant is willing and able to give informed consent for participation in the study
2. Male or female aged 50 years to 75 years
3. Current or Ex-smoker with at least 20 year pack history
4. or Less than 20 year pack history but with family history of lung cancer in a 1st degree relative (mother, father, sister, brother, child)
5. ECOG Status: 0, 1 and 2 (Eastern Co-operative Oncology Group)

**Grade**	**ECOG**
0	Fully active, able to carry on all pre-disease performance without restriction
1	Restricted in physically strenuous activity but ambulatory and able to carry out work of a light or sedentary nature, e.g., light house work, office work
2	Ambulatory and capable of all self-care but unable to carry out any work activities. Up and about more than 50% of waking hours

6. Geographical postal sectors of:

NHS Geographical Area	Eligible Postcodes
**Tayside**	DD1 - DD11 (except DD6), PH1–PH3 , PH6-PH8, PH10, PH11, PH13, PH15 & PH16
**Greater Glasgow & Clyde**	G1-G5, G11 –G15, G20-G22, G31-34, G40 –G46,<br><br>G51- G53, G60-G62 &G64, G66 & G69, G72 & G73,<br><br>G76-G78, G81-G83<br><br>PA1–PA8 (except PA6), PA11-PA16 & PA19

.

3.3 EXCLUSION CRITERIA

1. History of any cancer other than non-melanomatous skin cancer, cervical cancer in situ.
2. Symptoms suggestive of lung cancer within past 6 months (haemoptysis, unintentional weight loss (at least 5% in preceding 6 months).
3. Patients for whom the GP considers invitation to the study would cause undue distress.
4. Patients with other terminal disease.
5. Patients on prolonged / continuous use (> 3months) of cytotoxic/ immuno-suppressant drugs eg: Methotrexate, Cyclophosphamide, Azathioprine, Rapamycin, Mycophenolate, Rituximab and anti-immunophilins such as Ciclosporin, Tacrolimus. Monotherapy using glucocorticoids/ steroids eg prednisolone is NOT an exclusion criteria.

4 PARTICIPANT SELECTION AND ENROLMENT

4.1 IDENTIFYING PARTICIPANTS

Potentially eligible individuals will be identified from GP medical records by an electronic medical record search undertaken by the Scottish Primary Care Research Network. Potential participants will be recruited via their General Practitioner (through SPCRN) using a range of methods including:

a. postal invitation letter including a summary of the study Participant Information Sheet and a full PIS or Participant Information Brochure for those interested;

and, where necessary or appropriate:

b. invitation letter including a summary of the study Participant Information Sheet on collection of repeat prescription;

c. invitation during consultation with GP / Practice Nurse / Health Care Assistant at the practice;

d. invitation to those eligible on registered research volunteer databases

e. poster present in the GP's waiting room;

f. other recruitment strategies may be employed including; Media campaign involving: local and national newspapers; BBC Scotland; local radio,

Celebrity endorsement

g. Publicity campaign using posters/leaflets etc.…including:

Football/Bingo halls/ Bowling

Smoking Cessation Clinics

Hospital main entrances/ hospital clinics

Shopping Centres/Supermarkets/Pubs/etc.

Benefits offices/Post offices etc.

Sheltered Housing /Housing Associations

Community and charitable outreach programs

Mobile screening clinic

Pharmacist approach through practices.

The study invitation letter will include a slip for participants to either express interest in finding out more about the study, and provide their contact details or to request no further contacts about the study. Those returning an expression of interest will telephoned, more than 24 hours after anticipated receipt of the Participant Information Sheet/PIB, by a member of the research team. Additionally, the participant is given the opportunity to call or email the study team. The call (instigated by participant or study team) will allow a discussion of the study, to answer any questions the potential participant may have, do a preliminary assessment of eligibility and if they agree, to make an appointment for a recruitment visit (hereafter referred to as the eligibility assessment phone contact). An appointment letter/email will be sent out to confirm appointment.

A reminder call/email or text, whichever is preferable to the participant, will be carried our 2 days prior to the screening appointment. A reminder process decreases non-attendance.

Non-responders to the postal invite will be approached again, and for the last time, no longer than 2 months after the initial contact.

Where the recruitment rate falls short of the required number at each practice (170 per practice), practices will attach study invitation letters to repeat prescriptions of those aged between 50 years and 75. Those returning an expression of interest will be sent a full PIS/PIB and dealt with as above.

We will offer participating practices the option of using the Trial Torrent software package to identify potentially eligible patients who do not respond to the postal trial invite so that they can be approached during routine primary care consultations and given the study invite letter and Participant Information Sheet. Where potential participants express interest during the consultation, the software will notify the research team of that expression of interest. A member of the research team will undertake the eligibility assessment phone contact for those expressing interest in the consultation or by returning the expression of interest form at a later date. The recommended order (findings from focus groups) is;

- obtain consent
- take bloods from all consented participants (in the unlikely event; a blood sample is unobtainable or the blood sample blood sample from a participant in the test group is lost during transportation the participant will be contacted to arrange a subsequent sample.)
- complete survey questionnaire
- randomise to treatment arm

After randomisation group allocation is known all participates will be asked if they continue to be happy for their bloods to be used for the Early CDT- Lung Test

(lung cancer test group) and for future cancer related research for those who agreed by initialling the relevant box on the consent form.

For participants randomised to the intervention arm the EarlyCDT-Lung test will be performed and patients followed up according to their result.

At Visit 1 participants are advised that those with a positive EarlyCDT-Lung test result will be invited to a follow-up visit to interpret the test results and explain the progress on study thereafter. Those with a negative EarlyCDT-Lung test result will be

written to, explaining the test results and will be offered a follow-up visit or a telephone call if they wish. They will be advised of symptoms to watch for including persistent cough, coughing up blood, shortness of breath, weight loss or loss of appetite. They will be counselled to carry on having tests for other types of cancer if they are offered (e.g. bowel cancer test, mammograms, cervical smears).

Those in the control arm will be written to and thanked for their contribution to the study and advised and counselled identically to those with a negative test result.

All participants who agreed to donate blood for future will be advised that it will be used in cancer related research.

A patient specific section of the study website (www.eclsstudy.org) containing Participant Information Sheets and research staff contact details will be available for participants to view.

4.2 CONSENTING PARTICIPANTS

All individuals taking informed consent will have received training in Good Clinical Practice (GCP). It will be explained to patients that they are under no obligation to enter the trial and that they can withdraw at any time during the trial, without having to give a reason. A copy of the signed Informed Consent Form (ICF) will be given /or posted out to the study participant. A copy of the signed consent form will be given or sent to the GP with a letter outlining the study and patient pathway. The letter will notify the GP of their patients' group allocation, relevant Early CDT-Lung test result, and any notable findings found at the screening visit, namely, the request to give up smoking or relevant clinical information that requiring further clinical judgement. The original copy of the ICF is to be retained at the study site (ISF or TMF, as appropriate.). If any notable findings are found at the screening visit an anonymised copy of the GP letter will be filed in the participant's study file. If new safety information results in significant changes to the study risk–benefit assessment, the

Protocol, Participant Information Sheet and/or consent form will be reviewed, updated and amended as necessary. All participants will be informed of the new information, given a copy of the revised consent form and asked to re-consent if they choose to continue in the study.

4.3 SCREENING FOR ELIGIBILITY

SPCRN staff will visit practices to undertake searches of the GP computerised records. The resulting list of potentially eligible participants will be checked by a GP at the practice to ensure that those for whom study participation would cause undue distress and those with a terminal disease are not sent study invitations. Telephone screening of potentially eligible participants who have returned an expression of interest will be undertaken by a member of the research team at the eligibility assessment phone contact. This will include assessment of age, smoking history, family history of lung cancer, previous cancer, ECOG status and eligible postcode.

4.4 INELIGIBLE AND NON-RECRUITED PARTICIPANTS

The reason(s) for ineligibility will be explained to the patients and any questions they have will be answered. They will be thanked for their interest in the trial and any relevant clinical information will be communicated to their GP where the patient has given consent.

4.5 RANDOMISATION

Participants will be allocated to intervention or comparison group during the recruitment visit (Visit 1) using a web-based randomisation system; TRuST, provided by Tayside Clinical Trials Unit (TCTU). Set-up of the randomisation system will be by TCTU staff under the supervision of a TCTU statistician. Randomisation will be stratified by site and minimised by age, sex and smoking history

4.6 ADMINISTRATION OF THE TEST

Individuals at higher risk will be identified from GP medical records or community based recruitment as described above. Consenting individuals will be randomised to either receive an EarlyCDT-Lung test or standard care.

4.7 MANAGEMENT OF THE VISITS

Based on the test's 93% specificity and 41% sensitivity we anticipate that 400-450 participants in the intervention arm will have a positive test result. These will be offered a chest X-ray. Those with a negative or indeterminate X-ray will be referred for a CT scan. If the initial CT is negative then subsequent CTs will be offered 6 monthly for 24 months. Participants will receive appointments via mail/email (as preferred). Participants will be called 2-4 days before each CT-scan appointment. By calling, this allows the participant to ask any questions, check health status, arrange transport (if required) and increase participant retention. Individuals with monitorable abnormalities as classified by the radiology/respiratory physician's study panel on baseline CT scan or subsequent CT will be followed up over the study period or referred for NHS clinical care as appropriate. All individuals entering the study will be flagged and followed-up via the Scottish Cancer Registry. Participants who develop lung cancer will be individually followed-up via their medical records to assess both time to diagnosis and stage of disease at diagnosis. If no histological stage is available, stage will be assessed by a panel of three respiratory physicians blind to allocation status of the study subjects from chest X-rays or CT, or, if no imaging is available, medical assessment of stage will be carried out.

Prior to sending CT scan appointments participant deaths will be check using the SMR to ensure sensitivity is maintained. All participants in the EarlyCDT- Positive test groups known to have died will be removed from the CT scan appointment schedule register. If patients (EarlyCDT-Positive test) fail to attend for any imaging

assessment during the study, they will receive two reminders (one letter, one phone call). On the third non-attendance, a letter will be sent to the participant's GP to inform them of non-attendance. An appointment window of ± 4 weeks will be initiated for each scheduled CT scan.

Participants will receive results letters in relation to their initial CXR and CT scan and subsequent CT scans. Any clinical intervention/treatment will be arranged by the study team.

4.8 WITHDRAWAL AND STUDY TERMINATION PROCEDURES

No circumstances are anticipated for the withdrawal of patients from the trial initiated by the clinical team or trial investigators. Patients may choose to withdraw from the trial at any time, without giving a reason, and without compromising their future treatment.

If the study should be terminated early, for whatever reason, all participants with a positive Early CDT-Lung test will continue to be seen by the PI (lung specialist) and will continue to undergo any clinically relevant investigations and reviews and will be treated (if required) according to current clinical practice.

5 STUDY AND SAFETY ASSESSMENTS STUDY ASSESSMENTS

The main study assessment is the test result.

Other assessments include;

- diagnosis
- costs associated with intervention including any follow up/confirmatory tests and subsequent treatment
- costs associated with routine clinical management of patients

- health utility data
- mortality (various)
- measures of psychological outcomes and health behaviour
- other clinical outcomes uptake of subsequent investigations

5.1 SAFETY ASSESSMENTS

As the study does not employ an Investigational Medicinal Product, Adverse Events (AE) or Serious Adverse Events (SAE) will be recorded but not reported in the Annual Report. A number of factors affecting the trial population suggest that we would expect to observe a larger than normal incidence of episodes of ill-health due to both the age and co-morbidities of the study population. All known disease progressions and co-morbidities will be regarded as outcomes including complications arising from investigations which result in a hospital stay which will be captured in outcome 6 and all medical treatment or interventions will be predicated upon normal clinical care and not related to the study protocol. All CXR and CT scan incidental findings (incidentaloma) will be recorded in the CRF as an incidental finding and a specialist referral will be made as directed in a study SOP within the Study Operations Manual. AEs (as defined) will be recorded as soon as they are known either from the study subjects, PI patient review audits or via SMR or record review.

6 DATA COLLECTION & MANAGEMENT

6.1 DATA COLLECTION

It is the CI and PIs responsibility to ensure the accuracy of all data entered and recorded in the CRF/eCRFs and the database. The Delegation of Responsibilities Log will identify all trial personnel responsible for data collection, entry, handling and managing the database.

The data will be collected by the RN and/or the PI either directly onto a paper CRF with subsequent transcription to the eCRF, or direct data entry onto the web based eCRF. Where there is electronic storage of non-identifiable data this will be on a password protected device and/or database. A plan for data quality control will be developed by the data management staff at Tayside Clinical Trials Unit and the trial management team.

All research blood samples (anonymised using barcodes) will be labelled and packaged according to IATA regulations using Royal Mail Safeboxes or INTELSIUS or equivalent transport box systems to be transported to the University of Nottingham for processing, transporting and storage of samples for future research. All samples will be stored under custodianship as per UK Biobank guidelines. Sample Analysis and Chain of Custody Plans will be documented in the Study Operations Manual. The participant's medical notes (GP and hospital) paper or electronic will act as source data for relevant past medical history, subsequent medical conditions, hospital admissions and diagnostic reports.

Psychological and behavioural data relating to smoking, psychological outcomes including cancer worry, anxiety, depression and distress will be collected on all 10,000 participants through a baseline questionnaire administered during Visit 1. If required the RN can assist the participant with the completion of the questionnaire. Follow-up data will be collected between 1 and 24 months on subsets of the intervention and control groups. All participants in the EarlyCDT-positive group will be approached with the recruitment aim of 300 from this group collected at 1,3,6,12,18 and 24 months. The EarlyCDT-negative and control groups will be approached at the same rate as the EarlyCDT-positive group with the recruitment aim of 300 from each group, collected at 1,3,6 and 12 months.. A web-based tool will be used weekly to randomly sample patients from the EarlyCDT-negative and control groups, stratified by the two study centres. It is anticipated an average of 8 individuals (4 from each of

the two study centres) will be randomly sampled and invited to complete follow-up questionnaires from each of the EarlyCDT-negative and control groups per week (based on a 10 month recruitment period and an anticipated response rate of 67%). Response rates will be monitored and if they are lower than 67%, the number randomly sampled at each centre will be increased to achieve a minimum of 200 responses. Participants will be sent a £5 gift voucher for use in a range of stores for each questionnaire to be completed. A Cochrane review found the use of small monetary incentives significantly increases response rates to postal questionnaires (Edwards et al., 2009). There are precedents in trials and other UK studies where small monetary incentives have been used.

Two methods will be used for the initial period of recruitment: 50% of the sample will receive the questionnaire with the voucher and 50% will receive the voucher once they have returned their questionnaire. An assessment will be carried out to determine which of the two methods is more effective in maximising recruitment rate and will then be employed for the remainder of the study. .

A phone number will be provided for participants to call the research team for assistance in completing questionnaires. Occasionally the research team may call participants to check on postal delivery and offer assistance with completion to increase return rate. Participants who develop lung cancer during the 24 month follow-up period will not be sent further study questionnaires.

6.2 DATA MANAGEMENT SYSTEM

Tayside Clinical Trials Unit (TCTU) will provide a data management system using OpenClinica (https://www.openclinica.com/), its standard GCP-compliant data management system. Case Report Forms (CRF) will be developed together with the trial management team, statistician and data manager to ensure that the data management system supports the research aims of the study. The data management

system will be fully validated, including the provision of test data and supporting documentation. Data entry will be coordinated by TCTU. Data will be stored on servers controlled through the Tayside Medical Science Centre and housed within the Health Informatics Centre and the University of Dundee. Backup and disaster recovery will be provided by TCTU according to its standard operating procedures. The Statistical Analysis Plan will specify dummy tables linked to primary and secondary outcomes and the data management system will be designed to export directly to the dummy table formats for analysis.

7 STATISTICS AND DATA ANALYSIS

7.1 SAMPLE SIZE CALCULATION

The rate of lung cancer is 187/100,000 per year for patients aged 50-74 in Scotland 2008 (ISD cancer statistics). Deprivation is associated with a higher risk of lung cancer. Those in the most deprived quintile are associated with an increased risk of 1.8 times compared to the middle quintile of deprivation. (ISD cancer statistics) this gives an estimated annual lung cancer rate of 336/100,000 among the practices taking part in the study. A high risk group within this population will be selected using similar entry criteria (outlined above) as the Mayo screening study which had a 2% prevalence rate of lung cancer and a further 2% incidence rate over the following 5 years (Swensson 2005). The baseline rate of late stage presentation for the particular high risk population envisaged in this study is uncertain, as is the size of the reduction in late stage presentation likely to be achieved through use of EarlyCDT-Lung. Using an estimated late stage presentation rate of 1,200/100,000 per year in the control group i.e. 2.4% over the two-year follow-up period, we require 85% power at 5% significance (two-sided) to detect an estimated reduction of 35% in presentation rate in the test group i.e. as low as 780/100,000 per year or 1.56% over the two-year follow-up period. This corresponds to an estimated event rate over the

two years of follow-up of 120 events in the control group and 78 events in the test group and implies a required sample size of n=5,000 per group i.e. 10,000 altogether.

The anticipated 35% reduction in event rate between the control group and the test group is justified by current estimates of the capability of the EarlyCDT-Lung test to identify cases (41% sensitivity, 93% specificity) together with current estimates of the specificity of CT scanning (67%).

The sample size calculations are based upon standard methods for time to event data using the cpower function in R and stpower exponential procedure in Stata and assuming exponential survival. They were also confirmed using standard approaches for detecting a change in binomial probabilities, and confirmed using approaches to detect a change in Poisson rates (with essentially identical results as loss to follow up is expected to be low).

The study aims for a short recruitment period and so no allowance has been made for accrual. With such an allowance, say to 1 year, the power will increase to 91% to identify a 35% reduction provided the minimum follow up period of 2 years is observed.

For the follow-up analysis of behavioural and psychological outcomes, 200 participants in each group will allow a mean difference of 3.00 (SD 15.04 (unpublished data from the ProtecT prostate cancer study) in the Impact of Events Scale with 80% power and 2-sided 5% significance level. We will, however, collect data from 300 patients in each group to allow for attrition. Assuming 80% participants are current smokers, we will obtain 80% power at 5% significance level to detect a reduction in smoking from 80% to 67% i.e. 13% points difference assuming follow up on 200 participants.

7.2 PROPOSED ANALYSES

Characteristics of participants will be compared informally between treatment arms at baseline. The main analysis of the primary outcome will be intention-to-treat. Cox proportional hazards models will be used to estimate the hazard ratio of the rate of late stage lung cancer in the intervention arm compared to the control arm. Participants who are lost to follow up will be censored. The models will adjust for age, gender smoking history and practice. If appropriate, random cluster effects will be included rather than fixed effects for practices. A similar methodology will be used for the secondary outcomes of comparisons of mortality rates (secondary outcomes 3a and 3b). A subsequent analysis will compare the outcomes of those with EarlyCDT positive in comparison to those in the intervention group with EarlyCDT negative (primary contrast for this analysis) and those in the control group (secondary analysis 1). Comparisons of proportions (secondary analyses 1 and 4) will be carried out using chi square tests. Fishers exact test will be used if the numbers of events are small.

The analyses of the questionnaire responses (secondary analysis 5) will be carried out using the appropriate 2 sample t tests and regression methods at baseline. Non parametric tests will be used if there is evidence of non-normal scores. Multilevel models will be used to analyse the repeated scores during follow up.

Poisson regression models, adjusting for follow up time if necessary, will be used to investigate the other clinical measures (secondary outcomes 6 and 7).

7.2.1 Cost effective analysis

The short-term within-trial analysis will compare the costs and outcomes associated with the intervention group to those of the comparison group at 24 months. A longer term analysis will employ a decision analytic model to link the short term outcomes measured within the trial to potential longer term impacts on health (for example in

terms of impacts on the development of cardiovascular disease, diabetes etc.). Both analyses will utilise the NHS and personal social service perspective favoured by NICE.

7.3 MISSING DATA

The extent of missing data will be examined and, if necessary, methods such as multiple imputation will be implemented to provide robust results, assuming data are missing at random (MAR).

7.4 TRANSFER OF DATA

Transfer of Data will be achieved according to standard TCTU SOPs (Study Operations Manual).

7.5 PREGNANCY

The female age group in this study (50 to 75 years) areunlikely to be pregnant. However, assessment of risk is established when all women are asked about the possibility of pregnancy prior to any imaging investigations as per usual NHS risk assessment protocols.

8 TRIAL MANAGEMENT AND OVERSIGHT ARRANGEMENTS

8.1 TRIAL MANAGEMENT GROUP

The trial will be overseen by a Trial Steering Committee (TSC) and a Trial Management Committee (TMC) consisting of the CI and co-investigators, trial managers and with representation for research nurses and SPCRN. Day-to-day management of delivery of the trial will be achieved through the Trial Operations Group chaired by the Assistant Director of TCTU and comprising project and trial managers, data managers, statistician and software developers.

8.2 TRIAL MANAGEMENT

The Senior Trial Manager and Trial Manager will oversee the study and will be accountable to the CI. The Senior Trial Manager will be responsible for other trial processes hosted within the TCTU. However, this remains the overall responsibility of the CI. Any queries will be resolved by the CI or delegated member of the trial team.

A study-specific Delegation Log will be prepared for each site, detailing the responsibilities of each member of staff working on the trial.

8.3 TRIAL STEERING COMMITTEE

The TSC will be a mixture of lung cancer investigators and independent members. The TSC will be chaired by an independent, senior clinician with expertise in cancer research and clinical trials. Other independent members will include a statistician and a senior trial manager and a trial methodologist. The TSC will also have two patient representatives. The TSC will meet annually, with the first meeting being shortly after the start of the project. The terms of reference of the TSC and the draft template for reporting are detailed in Appendix 2. .

8.4 DATA MONITORING COMMITTEE

The DMEC will comprise of three independent members plus an independent statistician. The Chair will be a senior clinician or statistician with experience of clinical trials and DMECs. The two other independent members will be a senior trial manager or methodologist, together with a second clinical expert. The DMEC will meet annually in the first instance although the chair will be free to increase the frequency of meetings as required. The first DMEC will be held shortly after the first TSC.

The terms of reference of the DMEC are detailed in Appendix 3.

8.5 INSPECTION OF RECORDS

The CI, PIs and all institutions involved in the study will permit trial related monitoring, audits, REC review, and regulatory inspection(s). In the event of an audit, the CI agrees to allow the Sponsor, representatives of the Sponsor or regulatory authorities direct access to all study records and source documentation.

8.6 RISK ASSESSMENT

A pre-Sponsorship study risk assessment was carried out by the TASC Research Governance Manager prior to Sponsorship approval being granted.

8.7 STUDY MONITORING

The Sponsor will determine the appropriate extent and nature of monitoring for the study and will appoint appropriately qualified and trained monitors.

8.7.1 Potential Risks

8.7.2 Blood sampling

Veins and arteries vary in size from one patient to another and from one side of the body to the other. Obtaining a blood sample from some people may be more difficult than from others. Risks associated with having blood drawn are slight but may include:

- Excessive bleeding
- Fainting or feeling light-headed
- Hematoma (blood accumulating under the skin)
- Infection (a slight risk any time the skin is broken)

All research nursing staff will be highly trained and experienced in venipuncture thereby minimizing risk.

8.7.3 Test results

False positives and false negatives are explained as follows:

No medical test is completely accurate. This blood test is expected to pick up about 40 in 100 cases of lung cancer and detect the cancer at an early stage. However this means it doesn't pick up all cases of lung cancer. So even if your test is negative, or if you are in the non-test group, it is important that you see your GP if you are unwell in any way that could be due to lung cancer. This includes persistent cough, coughing up blood, shortness of breath, weight loss or loss of appetite.

As no medical test is completely accurate, the blood test will be positive in a small number of people who do not have early lung cancer. This will happen to about 11 out of every 100 people who have a positive test result. These people will be offered chest X-rays and lung scans to see if they have lung cancer. People with a negative blood test will not get any X-rays or scans.

8.7.4 Radiography

Risks relating to chest X-ray and CT scan are explained as follows:

Chest X-rays and lung scans use radiation. People can develop cancer because of this radiation, but this is very rare. The amount of radiation you get from a chest X-ray is very small. About 1 million people would need to have a chest X-ray for one extra person to develop cancer because of the chest X-ray. A CT lung scan gives about 600 times as much radiation as a chest X-ray. 1500 people would need to have a CT lung scan for one extra person to develop cancer because of the scan. These risks are very small compared to the one in four chance we each have of developing cancer in our lifetime. Only about 400-450 people in this study are expected to have a positive blood test and will, therefore, need chest X-rays and scans. The chances of radiation affecting anyone in this study in this way are therefore very small.

8.7.5 Minimising Risk

All associated risks are well understood and have established procedures for management.

9 GOOD CLINICAL PRACTICE

9.1 ETHICAL CONDUCT OF THE STUDY

The study will be conducted in accordance with the principles of good clinical practice (GCP) and the Research Governance Framework Scotland.

In addition to Sponsorship approval, a favorable ethical opinion will be obtained from an appropriate REC and appropriate NHS R&D approval(s) will be obtained prior to commencement of the study.

9.1.1 Confidentiality

All laboratory specimens, evaluation forms, reports, and other records will be identified in a manner designed to maintain participant confidentiality. All records will be kept in a secure storage area with limited access to study staff only. Clinical information will not be released without the written permission of the participant, except as necessary for monitoring and auditing by the Sponsor, its designee or Regulatory Authorities. The CI and study staff involved with this study will not disclose or use for any purpose other than performance of the study, any data, record, or other unpublished, confidential information disclosed to those individuals for the purpose of the study. Prior written agreement from the Sponsor or its designee will be obtained for the disclosure of any said confidential information to other parties.

9.1.2 Data Protection

The CI and study staff involved with this study will comply with the requirements of the Data Protection Act 1998 with regard to the collection, storage, processing and

disclosure of personal information and will uphold the Act's core principles. The CI and study staff will also adhere, if appropriate, to the current version of the NHS Scotland Code of Practice on Protecting Patient Confidentiality. Access to collated participant data will be restricted to the CI and appropriate study staff.

Computers used to collate the data will have limited access measures via user names and passwords.

Published results will not contain any personal data that could allow identification of individual participants.

9.1.3 Insurance and Indemnity

The University of Dundee and Tayside Health Board are Co-Sponsoring the study.

Insurance. –The University of Dundee will obtain and hold Professional Negligence Clinical Trials Insurance cover for legal liabilities arising from the study.

Tayside Health Board will maintain its membership of the Clinical Negligence and Other Risks Insurance Scheme ("CNORIS") which covers the legal liability of Tayside in relation to the study.

Where the study involves University of Dundee staff undertaking clinical research on NHS patients, such staff will hold honorary contracts with Tayside Health Board which means they will have cover under Tayside's membership of the CNORIS scheme.

Indemnity. The Co-Sponsors do not provide study participants with indemnity in relation to participation in the Study but have insurance for legal liability as described above.

10 STUDY CONDUCT RESPONSIBILITIES

10.1 PROTOCOL AMENDMENTS, DEVIATIONS AND BREACHES

The CI will seek approval for any amendments to the Protocol or other study documents from the Sponsor, REC and NHS R&D Office(s). Amendments to the protocol or other study docs will not be implemented without these approvals.

In the event that a CI needs to deviate from the protocol, the nature of and reasons for the deviation will be recorded in the CRF, documented and submitted to the Sponsor. If this necessitates a subsequent protocol amendment, this will be submitted to the Sponsor for approval and then to the appropriate REC and lead NHS R&D Office for review and approval.

In the event that a serious breach of GCP is suspected, this will be reported to the Sponsor immediately using the form “Notification to Sponsor of Serious Breach or Serious Deviation”.

10.2 STUDY RECORD RETENTION

To enable evaluations and/or audits from regulatory authorities, the investigators agree to keep records, including the identity of all participating patients (sufficient information to link records, all signed informed consent forms, source documents, and group allocation to intervention and control). The records should be retained by the study site coordinators and investigator according to TASC SOP or local NHS Board regulations, or as specified in the Clinical Study Agreement, whichever is longer.

If the CI, PI or a study site coordinator relocates, retires, or for any reason withdraws from the trial, the University of Dundee should be prospectively notified. The trial records must be transferred to an acceptable designee. The study site coordinator must comply with the TASC SOP on archiving and obtain written permission from the

Sponsor before disposing of any records, even if retention requirements have been met.

10.3 END OF STUDY

The end of study is defined as last patient last visit scan (LPLV) plus 24 M. The Sponsor, CI and/or the TSC have the right at any time to terminate the study for clinical or administrative reasons.

End of follow-up.

The end of the study will be reported to the Sponsor, REC and NHS R&D Offices within 90 days, or 15 days if the study is terminated prematurely. The CI will ensure that any appropriate follow up is arranged for all participants.

A summary report of the study will be provided to the Sponsor and REC within 1 year of the end of the study.

10.4 CONTINUATION OF TREATMENT FOLLOWING THE END OF STUDY

All participants will enter the standard NHS care pathway after their last scan; for further investigations or treatment if: a positive scan, classified nodules or incidental finding or if a non-referable scan is determined they will be monitored by their GP if they become symptomatic for lung cancer.

11 REPORTING, PUBLICATIONS AND NOTIFICATION OF RESULTS

11.1 AUTHORSHIP POLICY

Ownership of the data arising from this study resides with the study team. On completion of the study, the study data will be analysed and tabulated, and a clinical study report will be prepared.

Authorship eligibility for each manuscript arising from this study will be determined according to the criteria laid out in the Working Practice Document on Authorship filed in the Study Operations Manual.

11.2 PUBLICATION

The clinical study report will be used for publication and presentation at scientific meetings. Trial Investigators have the right to publish orally or in writing the results of the study.

Summaries of results will also be made available to trial Investigators for dissemination within their clinical areas (where appropriate and according to their discretion).

11.3 PEER REVIEW

This trial has undergone peer review by the Sponsorship Committee. The trial design and results will be reviewed in publications by the referees of the journal to which the paper (and its protocol) will be submitted.

12 REFERENCES/BIBILOGRAPHY

1. Aberle D, Adams A, Berg C, Black W, Clapp J, et al. Reduced Lung-Cancer Mortality with Low-Dose Computed Tomographic Screening. National Lung Screening Trial Research Team, New England Journal of Medicine 2011;365(5):395-409.

2. Bach, PB; Mirkin, JN; Oliver, TK; Azzoli, CG; Berry, DA et al Benefits and Harms of CT Screening for Lung Cancer. A Systematic Review. JAMA 307 22 2418-2429 DOI: 10.1001/jama.2012.5521

3. Bower P, Wilson S, Mathers N. How often do UK primary care trials face recruitment delays? Family Practice 2007; 24: 601–603

4. Boyle P, C. J. Chapman, S. Holdenrieder, A. Murray, C. Robertson, W. C. Wood Benefits and Harms of CT Screening for Lung Cancer. JAMA. 2012 May20, 1.2.

5. Boyle P, Chapman CJ, Holdenrieder S, Murray A, Robertson C, Wood WC, et al. Clinical validation of an autoantibody test for lung cancer. Annals of Oncology 2011;22(2):383-89.

6. Campbell MK, Snowdon C, Francis D, Elbourne D, McDonald AM, Knight R, Entwistle V, Garcia J, Roberts I, Grant A. Recruitment to randomised trials: strategies for trial enrolment and participation study. The STEPS study. Health Technology Assessment 2007; Vol. 11: No. 48

7. Chapman C, Robertson J, Murray A, Titulaer M, Lang B, Thorpe A, et al. The Presence of Autoantibodies to Tumour-Associated Antigens Can Predate Clinical Diagnosis of Small Cell Lung Cancer Chest 2010;138(No. 4 Supplement):775A.

8. Chapman CJ, et al. EarlyCDT®-Lung test: improved clinical utility through additional autoantibody assays. *Tumor Biology* 2012; DOI: 10.1007/s13277-012-0379-2.

9. Chou R, LeFevre ML. Prostate cancer screening--the evidence, the recommendations, and the clinical implications. JAMA. 2011 Dec 28;306(24):2721-2. doi: 10.1001/jama.2011.1891.

10. Edwards PJ, Roberts I, Clarke MJ, DiGuiseppi C, Wentz R, Kwan I, Cooper R, Felix LM, Pratap S. Methods to increase response to postal and electronic questionnaires. Cochrane Database of Systematic Reviews 2009, Issue 3. Art. No.: MR000008. DOI: 10.1002/14651858.MR000008.pub4.

11. Finn OJ. Immune Response as a Biomarker for Cancer Detection and a Lot More. New England Journal of Medicine 2005;353(12):1288-90.

12. Hardcastle J, Chamberlain J, Robinson M, Moss S, Amar S, Balfour T, et al. Randomised controlled trial of faecal-occult-blood screening for colorectal cancer. Lancet 1996;348:1472-7.

13. Hewitson P, Glasziou PP, Irwig L, Towler B, Watson E. Screening for colorectal cancer using the faecal occult blood test, Hemoccult. Cochrane Database of Systematic Reviews, 2007 Issue 1.

14. Lam S, Boyle P, Healey GF, Maddison P, Peek L, Murray A, et al. EarlyCDT-Lung: An Immunobiomarker Test as an Aid to Early Detection of Lung Cancer. Cancer Prevention Research 2011;4(7):1126-34.

15. Macdonald IK, Allen J, Murray A, Parsy-Kowalska CB, Healey GF, et al. (2012) Development and Validation of a High Throughput System for Discovery of Antigens for Autoantibody Detection. PLoS ONE 7(7): e40759. doi:10.1371/journal.pone.0040759

16. MacMahon H, Austin JHM, Gamsu G, Herold CJ, Jett JR, Naidich DP, et al. Guidelines for Management of Small Pulmonary Nodules Detected on CT Scans: A Statement from the Fleischner Society1. Radiology 2005;237(2):395-400.

17. Mathew J, Healey G, Jewell W, Murray A, Chapman C, Peek L, et al. Demographics of populations at high risk of lung cancer and results of the Early CDT-Lung test. Journal of Clinical Oncology 2010;28(no. 15_suppl):7033.

18. Mayor S. Critics attack the new NHS breast screening leaflets for failing to address harms. BMJ2010;341:c7267.

19. McCartney M. Doctors should stop supporting unethical screening. BMJ 2011;343;d4592.

20. Mirkin JN. Benefits and Harms of CT Screening for Lung Cancer. A Systematic Review

21. Murray A, Chapman CJ, Healey G, Peek LJ, Parsons G, Baldwin D, et al. Technical validation of an autoantibody test for lung cancer. Annals of Oncology 2010;21(8):1687-93.

22. Oken, M.M., Creech, R.H., Tormey, D.C., Horton, J., Davis, T.E., McFadden, E.T., Carbone, P.P.: ECOG Performance Status ref Toxicity And Response Criteria Of The Eastern Cooperative Oncology Group. Am J Clin Oncol 5:649-655, 1982.)

23. Robertson J, Chapman C, Cheung K, Murray A, Pinder S, Price M, et al. Autoantibodies in early breast cancer. Journal Clinical Oncology 2005;23 No. 16S, Part I of II (June 1 Supplement)(2005 ASCO Annual Meeting Proceedings):549.

24. Scottish Primary Care Research Network. Scottish School of Primary Care - SPCRN. http://www.sspc.ac.uk/spcrn/: Last accessed 10.1.12.

25. Spitz MR, et al. A risk model for prediction of lung cancer. *J Natl Cancer Inst* 2007; 99(9):715-726.

26. Swensen SJ, Jett JR, Hartman TE, Midthun DE, Mandrekar SJ, Hillman SL, et al. CT Screening for Lung Cancer: Five-year Prospective Experience1. Radiology 2005;235(1):259-65.

27. Tan EM. Autoantibodies as reporters identifying aberrant cellular mechanisms in tumorigenesis. The Journal of Clinical Investigation 2001;108(10):1411-15.

28. Treweek S, Pearson E, Smith N, Neville R, Sargeant P, Boswell B, et al. Desktop software to identify patients eligible for recruitment into a clinical trial: using SARMA to recruit to the ROAD feasibility trial. Informatics in Primary Care 2010;18(1):51-58.

29. Treweek S, Pitkethly M, Cook J, Kjeldstrøm M, Taskila T, Johansen M, Sullivan F, Wilson S, Jackson C, Jones R, Mitchell E. Strategies to improve recruitment to randomised controlled trials. Cochrane Database of Systematic Reviews 2010, Issue 4. Art. No.: MR000013. DOI: 10.1002/14651858.MR000013.pub5.

30. US Preventive Services Task Force. Screening for breast cancer: recommendation statement. Ann Int Med2009;151:716-26

31. Vedhara K, Kendrick D. Early Cancer Detection Test: Maximising Recruitment. University of Nottingham Report August 2012

32. Zhong L, Coe SP, Stromberg AJ, Khattar NH, Jett JR, Hirschowitz EA. Profiling Tumor-Associated Antibodies for Early Detection of Non-small Cell Lung Cancer. Journal of Thoracic Oncology 2006;1(6):513-19.

33. Edwards PJ, Roberts I, Clarke MJ, Diguiseppi C, Wentz R, Kwan I, et al. Methods to increase response to postal and electronic questionnaires. Cochrane Database Syst Rev 2009(3)

34. British Journal of Cancer (2013), 1–5 | doi: 10.1038/bjc.2013.1

APPENDIX 1: PREPARATORY FOCUS GROUP WORK

1.1 PROTOCOL

Research Protocol

Maximising recruitment in early cancer detection trials: The lung cancer trial

Version 1: 30/4/12

Investigators:
Professor Kavita Vedhara (IWHO, University of Nottingham)
Professor Denise Kendrick (Primary Care, University of Nottingham)
Professor John Robertson (Division of Breast Surgery, University of Nottingham)
Dr Roshan Das Nair (NUH NHS Trust & University of Nottingham)
Dr Kate Skellington-Orr (KSO Research Limited, Glasgow)
Professor Frank Sullivan (Population Health Sciences, University of Dundee)

Background

Approximately two thirds of trials fail to reach their recruitment target or have to extend their recruitment period (1,2). Failing to fulfil recruitment targets rates leads to underpowered studies, reduced generalisability, increased costs and delays in the implementation of effective interventions. Maximising recruitment is thus key to a trial's success.

Funding has been obtained for a large trial of the effectiveness and cost effectiveness of early cancer detection test in lung cancer, which is to be evaluated in individuals at high risk of lung cancer. The trial will be undertaken in general practices from disadvantaged areas in Glasgow and Dundee, commencing October 2012. Recruitment to trials amongst disadvantaged populations can be particularly challenging due to a lack of trust, limited knowledge of research and low literacy amongst potential participants (3). Previous research has demonstrated that qualitative methods can be used to inform recruitment strategies by tailoring recruitment to the trial population. For example, the ProtecT feasibility study, for prostate specific antigen testing for prostate cancer, explored men's views of trial participation, interpretation of study information, understanding and acceptance of randomisation and treatment. Findings fed into recruitment strategies and this resulted in the proportion of men consenting to randomisation increasing from 49% to 70% (4).

The current research has been designed to deliver tailored recruitment strategies and materials for the population to be targeted in the forthcoming early lung cancer detection trial. We propose to achieve this be addressing the following aims:

Aims

1. To explore potential trial participant views on:
   (a) issues likely to influence recruitment into the trial and willingness to be randomised (e.g., recruitment strategies; understanding of risk information; clinical equipoise and randomisation)
   (b) recruitment and study documentation (e.g., invitation letter, questionnaires);
   (c) factors which facilitate and hinder trial participation.
2. To develop recruitment processes and materials for use in this, and subsequent trials.

3. To contribute to the literature on methods for enhancing trial recruitment

Methods
We will be working with a local research company based in Scotland (KSO Research Limited) who will identify eligible participants; undertake the focus groups and complete the transcribing and analysis of material obtained through focus group discussions. As the clinical trial will recruit patients from both Glasgow and Dundee, we will be seeking to conduct 2 focus groups in each city, with up to 10 participants in each group.
Participants: The population to be targeted in the trial will be individuals at high risk of developing lung cancer aged between 50-75 years i.e., individuals who self-identify as current or former cigarette smokers with at least 20 pack-years, or a history of cigarette smoking plus family history of lung cancer which gives an individual a personal risk similar to a smoking history of 20 pack years).
Recruitment: KSO Research limited will use 'on-street' recruitment methods to recruit participants into the focus groups. This will involve a trained recruiter working in each of the two areas. The recruiter will target local amenities where eligible participants may be found, for example, smoking areas outside of recreational facilities, at local train or bus stations, etc. Recruitment will take place at different times/days over one week. The company have used this kind of 'on-street' recruitment before with considerable success. They have observed that finding people who agree 'at random' to participate are often more likely to show genuine commitment to participation than those who respond to press advertisements. Furthermore, meeting the recruiter face-to-face encourages an early relationship and opportunity for participants to ask any questions that they have about participation on the spot before agreeing to take part. This approach also has the advantage of being cheaper than a press advertisement with no risk of over-subscription. In accordance with usual practice, the local police will be notified of the recruiters' on-street presence and will be done with their support.

Individuals will be approached on the street at random, although efforts will be made to recruit a mix of genders in each area and to fill quotas in three separate age bands (50-59, 60-69, 70-75 years). The recruiter will first introduce themselves; provide a brief verbal introduction to the research and will enquire whether the individual would be willing to discuss a research project about a new blood test for lung cancer involving people who currently smoke or who have smoked previously (see recruitment questionnaire). Those who consent to further conversation will be asked first about their smoking status and family history of lung cancer to establish eligibility. Those who are not eligible will be thanked for their time and will only be given further information about the research should this be requested. Those who are eligible will be asked additional brief screening questions regarding age and working status. Individuals who agree to participate immediately will receive a participant cover letter and information sheet (enclosed) and will be asked to provide contact information so that they can be re-contacted and reminded of the date, time and location of the focus group. Participants who wish to consider the invitation first, will be provided with contact details for the research team and a participant information sheet. Individuals will be advised that they will receive £30 cash to thank them for their time and to cover their out of pocket and travel expenses. Anonymised data will be provided to the research team on:

- The number of recruitment sessions undertaken
- The number of people approached
- The number of those approached who were eligible to participate and reasons for ineligibility
- The number of those eligible who agreed to take par

- The reasons why people chose not to take part
- Characteristics of those who were eligible who did and did not agree to take part and the reasons for non participation (in an EXCEL spreadsheet)
- The number of those agreeing to take part who attended each focus group

Procedure: Written informed consent (enclosed) will be obtained from all participants prior to commencing the focus groups. Participants will be reminded that the discussions of the group are to be audio-taped and transcribed verbatim, but that they will not be identified in the recordings and, as such, their contributions to the discussions will remain anonymous. Participants will be asked to discuss issues related to the aims outlined in 1a-c above, with discussions structured according to a topic guide (enclosed). Participants will receive £30 at the end of the focus group to thank them for their time and to cover out of pocket and travel expenses and will be asked to sign a receipt for this.
Analysis: Audio recordings of focus groups will be transcribed verbatim and a thematic analysis will be undertaken to provide a rich and detailed account of the data (5). Strategies for ensuring quality assurance of credibility, transferability, dependability, and confirmability of analysis will be followed (6). The information from this analysis will be used to further refine the recruitment strategy and materials for the main trial. All original data files will be confidentially destroyed and written data stored anonymously.

References:
(**1**) Watson JM, Torgerson DJ. Increasing recruitment to randomised controlled trials: a review of randomised controlled trials. *BMC Med Res Method* 2006, *6*:34 doi:10.1186/1471-2288-6-34.
(**2**) McDonald AM, et al. What influences recruitment to randomised controlled trials? A review of trials funded by two UK funding agencies. *Trials* 2006, *7*:9 doi:10.1186/1745-6215-7-9.
(**3**) Lovato LC, et al. Recruitment for controlled clinical trials: literature summary and annotated bibliography. *Control Clin Trials* 1997;18(4):328-52.
(**4**) Donovan J, et al. Prostate Testing for Cancer and Treatment (ProtecT) feasibility study. *Health Technol Assess* 2003;7(14):1-88.
(**5**) Braun V, Clarke V. Using thematic analysis in psychology. *Qualitative Research in Psychology* 2006 3: 77-101.
(**6**) Shenton A. Strategies for ensuring trustworthiness in qualitative research projects. *Education for Information* 2004; 22: 63-75.

1.2 APPROVAL

Institute of Work, Health & Organisations
http://www.nottingham.ac.uk/iwho

The University of Nottingham
UNITED KINGDOM · CHINA · MALAYSIA

Kavita Vedhara

Faculty of Medicine & Health Sciences
School of Community Health Sciences
Institute of Work, Health & Organisations
The University of Nottingham
International House
Jubilee Campus
Nottingham
NG8 1BB
t: +44 (0)115 9515315
f: +44 (0)115 846 6625

25/05/2012

Dear Kavita

I-WHO Ethics Committee Review

Thank you for submitting your proposal on "Maximising recruitment in early cancer detection trials: The lung cancer trial". This proposal has now been reviewed by I-WHO's Ethics Committee to the extent that it is described in your submission.

I am happy to tell you that the Committee has found no problems with your proposal. If there are any significant changes or developments in the methods, treatment of data or debriefing of participants, then you are obliged to seek further ethical approval for these changes.

We would remind all researchers of their ethical responsibilities to research participants. The Codes of Practice setting out these responsibilities have been published by the British Psychological Society. If you have any concerns whatsoever during the conduct of your research then you should consult those Codes of Practice and contact the Ethics Committee.

You should also take note of issues relating to safety. Some information can be found in the Safety Office pages of the University web site. Particularly relevant may be:
The *Safety Handbook*, which deal with working away from the University.
http://www.nottingham.ac.uk/safety/
Safety circulars: Fieldwork P5/99A on http://www.nottingham.ac.uk/safety/fieldwork.htm
Overseas travel/work P4/97A on http://www.nottingham.ac.uk/safety/overseas.htm
Risk assessment on http://www.nottingham.ac.uk/safety/risk-assessment.htm

Responsibility for compliance with the University Data Protection Policy and Guidance lies with all researchers.

Ethics Committee approval does not alter, replace or remove those responsibilities, nor does it certify that they have been met.

We would remind all researchers of their responsibilities:
- to provide feedback to participants and participant organisations whenever appropriate, and
- to publish research for which ethical approval is given in appropriate academic and professional journals.

Yours sincerely

pp Neil Coulson

Professor Nadina Lincoln
Chair IWHO Ethics Committee

1.3 FINAL REPORT
SEPARATE DOCUMENT (attached)

APPENDIX 2. TRIAL STEERING COMMITTEE

TRIAL STEERING COMMITTEE

A Trial Steering Committee (TSC) should be set up with the following terms of reference:

Terms of Reference

1. To monitor and supervise the progress of the trial towards its interim and overall objectives;

2. To review at regular intervals relevant information from other sources (e.g. other related trials);

3. To consider the recommendations of the Data Monitoring and Ethics Committee (DMEC);

4. To advise on publicity and the presentation of all aspects of the trial.

Membership of TSC

The membership should be limited and include an independent Chairman (not involved directly with the trial other than as a member of the TSC), two or more other independent expert members and the Principal Investigator. Where possible the membership should include a lay/consumer representative. The trial coordinator, trial statistician, etc, should attend meetings as appropriate. Observers from the Host Institution may be invited to all meetings.

Guidance Notes

Meetings

Before the trial starts, the PI should organise a meeting of the TSC to finalise the protocol. The TSC should then meet at least annually, although there may be periods when more frequent meetings are necessary. Meetings should be organised by the PI. Papers for the meeting should be circulated in advance. An accurate minute should be prepared by the PI and agreed by all the members.

Trial Steering and Management

The role of the TSC is to provide overall supervision of the trial. In particular, the TSC should concentrate on the progress of the trial, adherence to the protocol, patient safety and consideration of new information. Day-to-day management of the trial is the responsibility of the PI. The PI may wish to set up a separate Trial Management Group to assist with this function.

Good Clinical Practice

The TSC should endeavour to ensure that the trial is conducted at all times to the standards set out in the MRC Guidelines for Good Clinical Practice (GCP).

Patient Safety

In all the deliberations of the TSC the rights, safety and well-being of the trial participants are the most important considerations. The TSC should ensure that freely given informed consent is obtained from each trial participant. The TSC should

advise the investigators on the completeness and suitability of the patient information provided.

Progress of the Trial

It is the role of the TSC to monitor the progress of the trial and to maximise the chances of completing the trial within the agreed time scale. At the first TSC meeting, targets for recruitment, data collection, compliance, etc, should be agreed with the PI. Based on these targets, the TSC should agree a set of data that should be presented at each meeting (see template).

The PI is required to submit an annual report to HTA. This report should be endorsed by the TSC, should stand alone, and contain sufficient information to enable HTA to assess the progress of the trial without the need to refer back to the original application. The annual report should inform HTA of any new information that has a bearing on safety or ethical acceptability of the trial or any significant complaints arising, with a justification of the decisions taken.

The DMEC should be asked to advise the TSC, and may be required to provide information on the availability of data collected to date (from this and other studies) and advice on the likelihood that continuation of the trial will allow detection of an important effect. This should be done using methods that do not unblind the trial.

Adherence to Protocol

The full protocol should be presented and agreed at the first TSC meeting. Any subsequent changes to the protocol must be approved by the TSC, LREC/MREC (and by HTA).

Data Monitoring and Ethics Committee

At its first meeting, the TSC should establish a Data Monitoring and Ethics Committee (DMEC) that meets regularly to review the data and results of any interim analyses.

Members of the DMEC should be independent of both the trial and TSC.

Consideration of New Information

The TSC should consider new information relevant to the trial including reports from the DMEC. It is the responsibility of the PI, the Chairman and other independent members to bring results from other studies that may have a direct bearing on future conduct of the trial to the attention of the TSC.

On consideration of this information the TSC should recommend appropriate action, such as changes to the protocol, additional patient information, or stopping the trial. The rights, safety and well-being of the trial participants should be the most important consideration.

It is the responsibility of the PI to notify the TSC, DMEC and relevant regulatory authority (if applicable) immediately of any unexpected serious adverse events occurring during the course of the trial.

Template for Trial Steering Committee Agendas and Reports

The TSC should meet at least once a year and compose an annual report.

The table below outlines the information that should be provided by the PI at each meeting. This template should be used as a basis for the agenda of TSC meetings and a template for the annual report. These headings may not be appropriate at every stage of an individual trial, or for all trials.

Name of Trial and Grant Number

Please include a graph plotting the cumulative target and achieved recruitment numbers against time since start of recruitment

Table

	**Target (date set)**	**Achieved (date)**
Sample size sought		
Date recruitment started		
Proposed date for end of recruitment		
Actual recruitment rate versus target rate (by month/ quarter)		
Acceptance rate, as a proportion (i) of those invited to participate		
(ii) of all eligible participants, if known		
Quarterly/monthly forecasts of recruitment for the planned remainder of the trial		
Losses to followup (i) as a proportion of those entered		
(ii) per month/ quarter		
Number still being followed up successfully and number who have completed followup		
Completeness of data collected		
Any available results (pooled)		

Any organisational problems		
Issues specific to the trial (as specified by the TSC)		

APPENDIX 3. DATA MONITORING COMMITTEE
DATA MONITORING & ETHICS COMMITTEE (DMEC)

The Data Monitoring & Ethics Committee (DMEC) is established to safeguard the interests of patients participating in randomised controlled trials.

The terms of reference and membership are based on the Medical Research Council Guidelines for Good Clinical Practice In Clinical Trials (1998).

The DMEC is the only body involved in the trial that has access to the unblinded comparative data. The role of committee members is to monitor these data and make recommendations to the Trial Steering Committee (TSC) whether there are any ethical or safety reasons why the trial should not be continued.

The Chair of the TSC should be made aware of all communication between DMEC and the Chief Investigator (CI).

The membership of the DMEC will incorporate a pool of statisticians, clinicians/epidemiologists, consisting of at least three members to represent clinical, statistical and clinical trial expertise.

All members of the group should be independent of the trial they are monitoring. The frequency with which the DMEC subgroup meets will be dependent on the needs of the individual trial. The CI should submit a detailed plan for the interim analysis before the trial commences. The plan must satisfy members of the DMEC group.

Communication between the CI and the DMEC Chair is encouraged but should not bypass the TSC Chair. The CI and the Chair of the TSC will agree with their DMEC group Chair a timely mechanism for reporting to the DMEC group. With the help of the trial statistician, the CI must provide blinded data, in strict confidence, to the DMEC group as frequently as the members of the subgroup request. Serious adverse events must be reported to the lead clinician of the DMEC group and chairperson of the relevant research ethics committee immediately. If appropriate, the Medicines Control Agency must also be informed of all serious adverse events. The template for reporting interim data should be used by all the PIs.

The DMEC group will discuss the data on adverse events and, if appropriate, efficacy data, either in a meeting or by teleconference. If necessary, they may request further data from the CI and trial statistician. In the light of interim data, and other evidence from relevant studies (including updated overviews of the relevant randomised controlled trials), the DMEC group will inform the TSC if, in their view, the trial should proceed or be terminated. They may also advise the TSC on modification of the protocol.

Unless cessation of the protocol is recommended by the DMEC, the TSC and collaborators and administrative staff will remain ignorant of the results of the interim analysis of efficacy and toxicity. Collaborators and all others associated with the study, may write to the DMEC, to draw attention to any concerns they may have about the possibility of harm arising from the treatment under study, or about any other matters that may be relevant.

Terms of Reference

1. To set up and maintain direct communication with the CI and Chair of the TSC. The Chair of the TSC should be made aware of all communication between the CI and DMEC group.

2. To receive a copy of the trial protocol and plans for interim analysis prior to commencement of the trial, or, in the case of the first wave of trials, as early as possible.

3. To receive reports (as per template) during the trial at intervals agreed with the TSC and CI. It would be expected that these would be 6 monthly in the first year, and no less frequent than 12 monthly after that.

4. If interim analysis of the trial data is not planned in the protocol the subgroup should determine whether interim analysis should be undertaken.

5. To consider data from interim analyses, unblinded if considered appropriate, plus any additional safety issues for the trial and relevant information from the template and other sources.

6. In the light of 3., 4. and 5., and ensuring that ethical considerations are of prime importance, to report to the TSC and recommend on the continuation of the trial.

Output and Reporting by DMEC group

Format of First Meeting

1. The first meeting of the group will generally be an open meeting with the trial investigators (CI and trial statistician). The output of that meeting will include agreement on the relevant material for that particular trial which needs to be reported subsequently within the template.

2. The report of the trial statistician to the DMEC group will be seen only by the group members. Each group meeting should be summarised in the form of brief minutes. These minutes and the report of the trial statistician will be circulated only to the DMEC group members.

3. A very brief summary of the recommendations of each subgroup meeting should be sent to the Chairman of the TSC.

DATA MONITORING AND ETHICS COMMITTEE

REPORT TEMPLATE 1

Trial Summary and Analysis Plan for Pre-trial Submission

1. Title:
2. Grant No:
3. Principal Investigator:
4. Introduction to the trial
    4.1 Background in brief:
5. Methods in Brief
    5.1 Design of the trial
    5.2 Details of interventions
    5.3 Outcome measures
        Primary:
        Secondary:
    5.4 Eligibility criteria
        Inclusion Criteria:
        Exclusion Criteria:
    5.5 Sample size & Analysis
6. Baseline Characteristics that will be analysed for internal and external validity

Internal Validity

(Comparability between the treatment groups)

External Validity

(Comparability between trial participants and non-participants)

(Comparability between high and low recruiting centres)

All serious adverse events, as defined below, must be report to the lead clinician of the DMEC monitoring subgroup and chairperson of the ethics committee as soon as possible.

Any untoward medical occurrence that at any dose:

- Results in death
- Is life threatening
- Requires in-patient hospitalisation or prolongation of existing hospitalisation
- Results in persistent or significant disability/incapacity
- Is a congenital anomaly/birth defect

DATA MONITORING AND ETHICS COMMITTEE

REPORT TEMPLATE 2

DMEC Report Date:

Report Number:

1. Title:

2. Trial Progress

2.1 Trial recruitment

2.1.1 Plan of recruitment

Start date of recruitment =

End date of recruitment =

Recruitment period =

Expected average monthly recruitment =

Recruiting centres =

2.1.2 Recruitment to date

Recruitment period to date =

Total recruitment to date =

Observed average monthly recruitment =

Recruitment stratified by centre =

Expected recruitment period
(based on current recruitment rate) =

End date of recruitment
(based on expected recruitment patterns) =

Please insert a graph showing the planned and observed recruitment rates

2.1.3 Recruitment based on eligibility

Inclusion/exclusion

Number ineligible

Non-consent

Protocol violation

2.2 Internal Validity

Comparability of selected baseline characteristics
between the treatment groups

2.3 External Validity

2.3.1 Selected baseline characteristics of
trial participants and non-participants

2.3.2 Selected baseline characteristics of subjects
in high and low recruiting centres

2.4 Protocol Compliance

2.4.1 Number of patients withdrawn from treatment but continued being followed up

2.4.2 Number of patients who have been lost to follow up

2.4.3 Number of patients with missing follow up data

2.5 Frequency of primary events (if applicable)

3. Did you submit any data for interim analysis of efficacy

Yes/No

4. Analysis of safety data.

These are to be presented overall and by group:

4.1 Serious adverse events

These are defined as any untoward medical occurrence that at any dose:

- Results in death
- Is life threatening
- Requires in-patient hospitalisation or prolongation of existing hospitalisation
- Results in persistent or significant disability/incapacity
- Is a congenital anomaly/birth defect

To be summarised here but reported immediately to the Chairs of the DMEC subgroup and ethics committee and if appropriate to the Medicines Control Agency

4.2 Other adverse events

4.3 Abnormal laboratory tests (if applicable)

5. List any new publications on the safety and efficacy of the trial medications and provide copies.

6. List any new national or international guidelines on the treatment of the disease being studied and provide copies.
