# Supplementary material for: Five year mortality in an RCT of a lung cancer biomarker to select people for low dose CT screening
Source: PLoS One. 2025 Jan 8;20(1):e0306163. doi: 10.1371/journal.pone.0306163 (PMC11709295; doi:10.1371/journal.pone.0306163)
Supplement: Attachment — Submitted filename: Response to Reviewers.docx [file pone.0306163.s003.docx]

**Response to Reviewers**

**Editor’s requests**

1. Ensure that your manuscript meets PLOS ONE's style requirements, including those for file naming
   a. Done
2. The grant information you provided in the ‘Funding Information’ and ‘Financial Disclosure’ sections do not match
   a. Done
3. Include the following statement: ""This does not alter our adherence to  PLOS ONE policies on sharing data and materials.” (as detailed online in our guide for authors <http://journals.plos.org/plosone/s/competing-interests>
   a. Added to submission template and updated cover letter.
4. Include your updated Competing Interests statement in your cover letter
   a. Done
5. Include your full ethics statement in the ‘Methods’ section of your manuscript file. In your statement, please include the full name of the IRB or ethics committee who approved or waived your study, as well as whether or not you obtained informed written or verbal consent.
   a. Done
6. Please include captions for your Supporting Information files at the end of your manuscript, and update any in-text citations to match accordingly
   a. Done
7. Update title to reduce ambiguity
   a. Change made

**Reviewer 1**

8. Amend 1^st^ sentence of abstract
   a. Change made
9. Report the inclusion criteria for high-risk .
   a. Provided in references 24 and 25. If required a box containing the text in supplementary material section 9 could be added at the point on page 4 indicated in the text,
10. Clarify how the compliance to the protocol was defined
    a. Added to relevant section of text with greater detail in reference 24
11. Compare the outcomes in the model including a variable that classifies the participants in three groups according to randomization arm and test result.
    a. See section 11 of the supplementary material which deals with this point. Although this subgroup analysis may be of interest to readers, we have not included it in the main paper as the study is presenting the results of a trial comparing the Intervention V Control.
12. Report the results in the flow chart with yearly risk by arm & testing results.
    a. Hazard ratios have been added to the consort diagram or the table presented in the Supplementary material section

13.Provide data comparing the Hazard (ratio) in those who tested positive vs. the entire control arm.

b. See Supplementary table section 13

14 Explain the intervention: who underwent the test (people at high risk, heavy smokers

c. See response 9 above

15. Discussion should focus on the reliability and limits of the “per-protocol” analysis
    a. Changes made to paragraph 1 of the discussion to emphasise this point

**Reviewer 2**

16. Provide a table with estimates and standard errors where we do not only see the estimate of the treatment effect but also the coefficients of the confounding variables (age, gender, smoking history, and practice)
    a. Added as section 16 to supplementary material
17. Provide the models for when models converged
    a. Provided in response to 16 above
18. Provide details of Poisson regression analysis
    a. See supplementary material section 18
19. Change title of Outcomes section to Statistical analysis
    a. Done
20. Remove repeated sentence on p5
    a. Done
21. Report the results of the proportionality test as supplementary material.
    a. See Supplementary material section 21
22. Report p values on p7
    a. done

**Additional editorial requests**

23. Upload your figure files to the Preflight Analysis and Conversion Engine (PACE) digital diagnostic tool, [https://pacev2.apexcovantage.com/](https://eur01.safelinks.protection.outlook.com/?url=https%3A%2F%2Fpacev2.apexcovantage.com%2F&data=05%7C02%7Cfms20%40st-andrews.ac.uk%7C8f0dcff3e3b34e77a41f08dcafa94938%7Cf85626cb0da849d3aa5864ef678ef01a%7C0%7C0%7C638578390262995938%7CUnknown%7CTWFpbGZsb3d8eyJWIjoiMC4wLjAwMDAiLCJQIjoiV2luMzIiLCJBTiI6Ik1haWwiLCJXVCI6Mn0%3D%7C0%7C%7C%7C&sdata=OlCOhkQ2VoBqnAa9ypnQMsAU31N9HRzohfWD3FL%2FDWQ%3D&reserved=0)

Done though see point 12
